# Dynamics of SARS-CoV-2 seroassay sensitivity: a systematic review and modelling study

**DOI:** 10.2807/1560-7917.ES.2023.28.21.2200809

**Published:** 2023-05-25

**Authors:** Nana Owusu-Boaitey, Timothy W Russell, Gideon Meyerowitz-Katz, Andrew T Levin, Daniel Herrera-Esposito

**Affiliations:** 1 Case Western Reserve University School of Medicine, Cleveland, United States; 2 Centre for the Mathematical Modelling of Infectious Diseases, London School of Hygiene & Tropical Medicine, London, United Kingdom; 3 University of Wollongong, Wollongong, Australia; 4 Dartmouth College, Hanover, United States; 5 National Bureau for Economic Research, Cambridge, United States; 6 Centre for Economic Policy Research, London, United Kingdom; 7 Department of Psychology, University of Pennsylvania, Philadelphia, United States; 8 Laboratorio de Neurociencias, Universidad de la República, Montevideo, Uruguay; 9 Centro Interdisciplinario en Ciencia de Datos y Aprendizaje Automático, Universidad de la República, Montevideo, Uruguay; * These authors contributed equally to this work.

**Keywords:** SARS-CoV-2, COVID-19, Serology, Meta-analysis, Serosurveillance, Antibody, Serological assays

## Abstract

**Background:**

Serological surveys have been the gold standard to estimate numbers of SARS-CoV-2 infections, the dynamics of the epidemic, and disease severity. Serological assays have decaying sensitivity with time that can bias their results, but there is a lack of guidelines to account for this phenomenon for SARS-CoV-2.

**Aim:**

Our goal was to assess the sensitivity decay of seroassays for detecting SARS-CoV-2 infections, the dependence of this decay on assay characteristics, and to provide a simple method to correct for this phenomenon.

**Methods:**

We performed a systematic review and meta-analysis of SARS-CoV-2 serology studies. We included studies testing previously diagnosed, unvaccinated individuals, and excluded studies of cohorts highly unrepresentative of the general population (e.g. hospitalised patients).

**Results:**

Of the 488 screened studies, 76 studies reporting on 50 different seroassays were included in the analysis. Sensitivity decay depended strongly on the antigen and the analytic technique used by the assay, with average sensitivities ranging between 26% and 98% at 6 months after infection, depending on assay characteristics. We found that a third of the included assays departed considerably from manufacturer specifications after 6 months.

**Conclusions:**

Seroassay sensitivity decay depends on assay characteristics, and for some types of assays, it can make manufacturer specifications highly unreliable. We provide a tool to correct for this phenomenon and to assess the risk of decay for a given assay. Our analysis can guide the design and interpretation of serosurveys for SARS-CoV-2 and other pathogens and quantify systematic biases in the existing serology literature.

**Key public health message**

**What did you want to address in this study?**

Knowing how many people get infected with SARS-CoV-2 is important for determining the severity of the virus, herd immunity thresholds and groups at higher risk. To estimate this, results from antibody tests are used. However, antibody levels fall with time after infection, which can make these tests unreliable. We aimed to quantify the reliability of different tests, to understand possible biases in our understanding of COVID-19.

**What have we learnt from this study?**

The change of antibody test reliability through time is very variable, and it depends on assay characteristics. Some assays will give strongly biased estimates of infections a couple of months after an epidemic wave, while others will remain reliable for many months. We provide a tool for researchers to assess the risk that an assay will give biased results, and to quantitatively correct for this effect.

**What are the implications of your findings for public health?**

Because test reliability changes across time, some antibody tests have the potential to strongly bias our understanding of crucial aspects of COVID-19. As this effect varies depending on the test, the reliability of test results needs to be considered in a test-specific way. For future outbreaks and new infectious diseases, it is important that public health agencies provide guidelines and tools to account for this possible bias.

## Introduction

Throughout the COVID-19 pandemic, policymakers have been guided by the number of past infections inferred from serological assays. Seroassays have been heavily used to estimate the proportion of individuals that have been infected, the rate of fatal or severe infections [1-5] and population-wide immunity [6-8], and to anticipate the effect of future infection waves [9,10], among other purposes.

However, antibody levels wane with time after infection [11], reducing the sensitivity of serological assays for detecting previous infections [12-14]. We refer to the decay of assay sensitivity (in the context of serosurveillance) with time after seroconversion as seroreversion (by ‘time’, we refer to the time spanned between COVID-19 diagnosis and serological testing). Seroreversion is a major potential source of bias when estimating numbers of infections [1,15,16], and because these estimates guide public health policies such as vaccination programmes, it is important to account for this phenomenon.

More broadly, understanding seroreversion in general is important for the management of other emerging infectious diseases. For this, the study of severe acute respiratory syndrome coronavirus 2 (SARS-CoV-2) infections presents a unique opportunity. Firstly, an emergent pathogen with distinct symptoms, leading to a high rate of people seeking diagnosis and doctors requesting tests, and short incubation times allows for precise timing of epidemic waves and infections. Secondly, in some cohorts, it can be assumed that reinfections are rare (i.e. serosurveys performed after first epidemic waves). Thirdly, large numbers of serological surveys were performed for SARS-CoV-2 infection, using a wide range of assays and cohorts. These features of the COVID-19 pandemic allow for a rich analysis of seroreversion.

Strikingly, there is a lack of general analyses and guidelines to correct for seroreversion in the SARS-CoV-2 literature, to the best of our knowledge [15-17]. Time-varying sensitivity of seroassays has been evaluated in previous studies, but these were limited to few assays or short time spans [13,14,18-21]. Other studies analysed the change in quantitative antibody levels [11,13,22-26], which is informative for other uses of serological assays (e.g. studying immune protection), but not for infection surveys.

We performed a systematic review and meta-analysis of serology studies of COVID-19, to better characterise seroreversion across assays. We collected and curated time-specific sensitivity estimates from serological studies testing previously diagnosed COVID-19 patients who had not received COVID-19 vaccines. We analysed 76 of more than 400 screened studies, encompassing 50 seroassays, 290 data points and 44,992 tests.

We present time-varying sensitivity estimates for the assays included in the analysis and the dependence of seroreversion on assay characteristics. Finally, we compare time-varying sensitivities to manufacturer-reported sensitivities and estimate the risk of seroreversion bias in the literature, providing an overview of how seroreversion impacted the performance of emergency-approved seroassays during the COVID-19 pandemic.

## Methods

### Literature search

We performed a systematic literature review of seroprevalence studies, including studies identified up to 13 July 2022 and using search parameters detailed in a prior publication [27].

We supplemented this analysis with a search on medRxiv, BioRxiv, PubMed, SSRN and Google Scholar, on 30 June 2021 using the key “COVID-19 longitudinal, antibody waning” and on 15 February 2022 using the key “COVID-19 seroreversion”. Additional studies were taken from a prior review [28]. If a study cited prior publications assessing seroreversion in the same research cohort, we included those prior publications.

**Figure 1:**
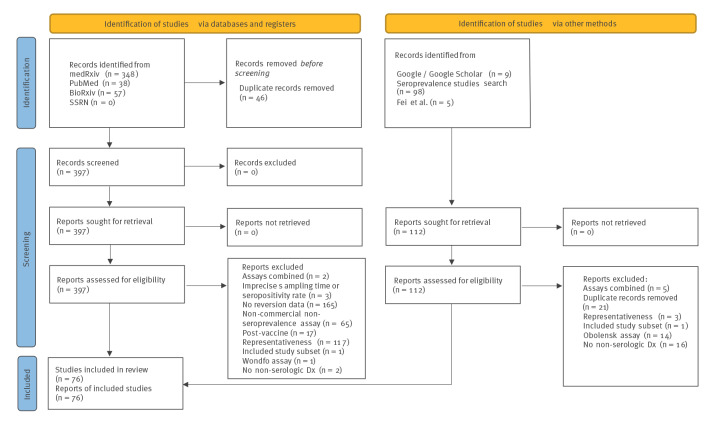
Prisma flow diagram, systematic review on SARS-CoV-2 seroassay sensitivity, January 2020–July 2022 (n = 555)

Inclusion and exclusion criteria for studies to be included in the analysis are listed in the Supplement, section A. The results of the systematic search are summarised in Figure 1. Broadly, we excluded studies reporting on vaccinated individuals and on highly unrepresentative groups. For the final list of included studies, see Supplementary Table S1. Details of the included study cohorts (e.g. age, sex) are shown in Supplementary Table S2 and further discussed in Supplement section A. Most cohorts (90%) were serologically tested during 2020, indicating that the reinfection incidence is likely to be low in the analysed data [29] and that infections mainly correspond to the original SARS-CoV-2 variant [30]. A list of the included studies and search details is presented in the GitHub repository associated with the project.

Dx: diagnosis; SARS-CoV-2: severe acute respiratory syndrome coronavirus 2; SSRN: Social Science Research Network.

Diagram from [41].

### Analysed assay characteristics 

Serological assays have different characteristics. We considered only some assay characteristics to keep model complexity low. We did not consider antibody isotype because IgG is used in all assays, and a preliminary analysis did not show effects for including other isotypes (data not shown). We considered whether the assay was quantitative or a lateral flow assay (LFA). We did not consider the specific type of quantitative readout technique, guided by preliminary analyses (data not shown). We considered all three antigens: nucleocapsid, spike protein and S1 receptor-binding domain (RBD). We considered three different types of antibody binding in quantitative assays: indirect, competitive and direct (the latter also called double-antigen sandwich assays in the literature).

### Statistical model

We fitted a hierarchical logistic regression Bayesian model to the data. For a given cohort of *N* serologically tested individuals in a study (all of whom had a previous COVID-19 diagnosis), we modelled the likelihood of the number of positive results *x*, with a binomial distribution with sensitivity *θ*:

$$P\left(x|\theta \right)\propto \theta^{x}{\left(1-\theta \right)}^{N-x}$$

Each cohort of *N* individuals tested in a study was associated with a time of testing *t* (i.e. the average time between COVID-19 diagnosis and serological testing for this cohort). Throughout the text, we refer to a cohort of individuals tested in a given study *s*, at a given time *t*, with an assay *a*, as a data point (e.g. a cohort tested across different times corresponded to multiple data points). We modelled the sensitivity of data point *θ_a,s,t_* (assay *a*, time *t*, study *s*) with the logit function:

$$log\left(\frac{\theta_{a,s,t}}{1-\theta_{a,s,t}}\right)=\left(\mu +u_{a}+u_{s}\right)+\left(\beta +b_{a}\right)\times t$$

where *μ* is the mean intercept, *u_a_* and *u_s_* are the random effects on the intercept of assay and study, *β* is the mean time-slope and *b_a_* is the random effect of assay on the slope. We set flat priors for *μ* and *β*. We set gamma priors with shape and rate parameters of 4 for the standard deviations *σ_ua_*, *σ_us_* and *σ_ba_* of the random effects.

To study the effect of assay characteristics, we modified the equation of the logistic regression to include their effects on the slope:

$$log\left(\frac{\theta_{a,s,t}}{1-\theta_{a,s,t}}\right)=\left(\mu +u_{a}+u_{s}\right)+\left(\beta_{LFA}L_{a}+\beta_{Direct}D_{a}+\beta_{Competitive}C_{a}+\beta_{Nucleocapsid}N_{a}+\beta_{Spike}S_{a}+\beta_{RBD}RBD_{a}+b_{a}\right)\times t$$

Parameters *β_LFA_*, *β_Direct_* and *β_Competitive_* are the effects on the time slope of using, respectively, LFA, quantitative-direct or quantitative-competitive assay designs. Variables *L_a_*, *D_a_* and *C_a_* take values of 0 or 1 to indicate whether assay *a* uses that design. We did not include an effect for the quantitative-indirect design, making it the baseline slope (thus, the parameters above indicate a difference relative to this design). Similarly, *β_Nucleocapsid_*, *β_Spike_* and *β_RBD_* are the effects of the antigen used on the time slope.

We fitted the models using STAN [31], with four chains with 4,000 draws each (1,000 warmup) and default parameters.

We tested the model fits using a cross-validation analysis, leaving out data points from model fitting and obtaining sensitivity predictions for the left-out data. We repeated this procedure to obtain an estimate for every data point. We used a tailored procedure that required that every prediction involved extrapolation of the model through time (for details see Supplement section B).

### Estimation of testing times

When studies did not report the median time between diagnosis and serological testing for their cohort, we estimated these times using reported case curves [32] for the study’s location (see details in Supplement section A).

### Data and code availability

All the data, code, literature pointers and review comments are available at the associated GitHub page.

## Results

### Assay variability in seroreversion

**Figure 2:**
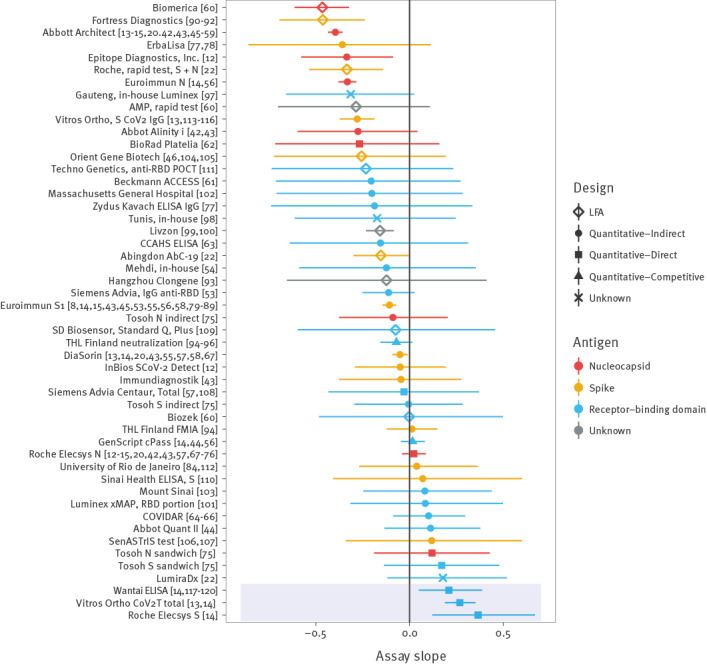
SARS-CoV-2 seroassay slopes estimated without assay characteristics, January 2020–March 2022 (n = 290 cohorts)

LFA: lateral flow assay; SARS-CoV-2: severe acute respiratory syndrome coronavirus 2.

We fitted a model without considering assay characteristics. In Figure 2, we see the slope of sensitivity decay obtained for each assay (we provide the corresponding sensitivity–time curves in Supplementary Figure S1). Estimated slopes were highly variable across assays (random effects of the assay were *σ_ua_* = 0.26 (95% credible interval (CrI): 0.19–0.36) for the intercept and *σ_ba_* = 0.66 (95% CrI: 0.31–1.04) for the slope). Interestingly, although most assays had decreasing sensitivity as expected (negative slopes), some assays had increasing sensitivities (positive slopes, shaded region in Figure 2). The positive slopes were not due to a lower starting sensitivity, or an initial increase followed by a decay. In Supplementary Figure S2, we provide an additional analysis in which an early and a late slope are fitted to these assays, where both early and late changes in sensitivity were increasing. There was also considerable variability in the intercepts between different studies using the same assay, with a standard deviation of *σ_us_* = 0.81 (95% CrI: 0.67–0.97) (larger than the between-assay standard deviation), outlining the importance of this source of variability.

Points show the mean of the posterior distribution of the slope for each assay; horizontal bars show the 95% credible intervals. The slopes were obtained from the model that does not include effects of assay characteristics, but colour and point shape indicate the characteristics of the assays. The grey shaded area marks assays with slopes significantly greater than 0. Sources: [8,12-15,18,20,42-120].

We note that while some assays had many data points spanning several months, other assays only had a few time points (several assays with only a few data points can be seen in Supplementary Figure S1). For the latter, our model’s sensitivity estimates involved extrapolation of sensitivity across time. We tested our model’s performance at extrapolation using a cross-validation procedure specifically designed for this (method details in Supplement section B). We found that the 95% CrI contained the validation data 91.7% of the time. For assays with fewer than nine data points (which applied to 99 of the 290 data points), 95.1% of the data points were within the cross-validation CrI.

### Assay characteristics determine seroreversion

Next, we analysed the relation between assay characteristics and sensitivity decay. We fitted a model with effects of different assay characteristics on the assay-specific slope. We included terms for each of the three antigens (nucleocapsid, spike, and RBD) and for three different assay designs (LFA, quantitative-direct, quantitative-competitive), leaving the fourth assay design (quantitative-indirect) as the baseline slope.

**Figure 3:**
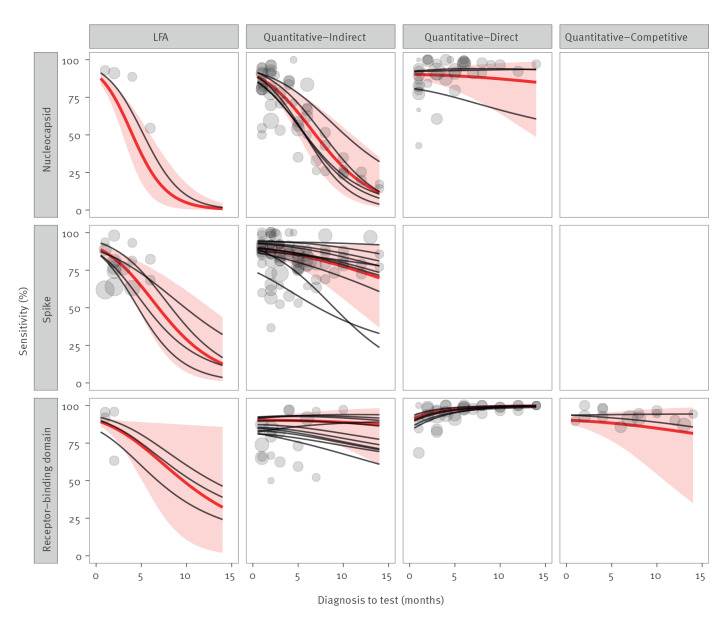
Sensitivity profiles for different SARS-CoV-2 seroassay characteristics, January 2020–March 2022 (n = 276 cohorts)

LFA: lateral flow assay; SARS-CoV-2: severe acute respiratory syndrome coronavirus 2.

Both the analytic technique and the antigen showed important effects on seroreversion (Figure 3, Table 1). The slope term for LFA assays was negative, *β_LFA_* = −0.23 (95% CrI: −0.40 to −0.07), and *β_LFA_ <* 0 in 99.6% of the posterior samples, indicating that their sensitivity decayed faster than that of quantitative-indirect assays. The slope term for quantitative-direct assays had a value of *β_Direct_* = 0.31 (95% CrI: 0.15–0.48), and *β_Direct_ >* 0 in 99.9% of the posterior samples, indicating that they decayed more slowly. The term for quantitative-competitive assays had a value of *β_Competitive_* = −0.03 (95% CrI: −0.25 to 0.20), and *β_Competitive_ >* 0 in 40.1% of the posterior samples, showing no clear difference compared with the quantitative-indirect assays (which may be due to the small number of assays in the quantitative-competitive group). Differences between analytic techniques can be appreciated by comparing the different columns of Figure 3.

The sensitivity profile across time for each kind of assay is shown in a different panel. Rows indicate the targeted antigen, and columns indicate the analytic technique. For example, the panel in the second row and second column shows assays that target the spike protein and use a quantitative-indirect design. Red lines: mean sensitivity for each kind of assay; shaded regions: 95% credible intervals of the mean sensitivity of the group (i.e. not accounting for variability between assays); black lines: fits for individual assays; grey dots: data, with size proportional to the square root of sample size. Empty panels indicate that no assays with those characteristics were found for the analysis.

**Table 1:** Estimated sensitivities of SARS-CoV-2 seroassays at each time after diagnosis, for each type of assay fitted in the analysis, January 2020–March 2022 (n = 276 cohorts)

Time after diagnosis (months)	Sensitivity by assay type in % (95% CrI)								
	LFA	LFA	LFA	Q-indirect	Q-indirect	Q-indirect	Q-direct	Q-direct	Q-competitive
	N	S	RBD	N	S	RBD	N	RBD	RBD
1	84 (79–89)	87 (83–91)	88 (84–91)	87 (83–91)	89 (86–92)	90 (87–93)	90 (87–93)	93 (90–95)	90 (86–93)
2	75 (66–83)	83 (76–88)	85 (78–91)	83 (77–88)	88 (84–92)	90 (87–93)	90 (85–94)	95 (92–96)	90 (85–93)
3	64 (49–76)	78 (67–86)	82 (71–90)	78 (69–85)	87 (82–92)	90 (86–94)	90 (84–94)	96 (94–98)	89 (82–94)
4	50 (32–69)	71 (57–83)	78 (61–89)	72 (60–82)	86 (80–91)	90 (84–94)	90 (82–95)	97 (95–98)	89 (80–95)
5	37 (19–60)	64 (45–80)	73 (51–89)	65 (50–78)	85 (77–91)	90 (83–95)	89 (80–95)	98 (96–99)	88 (76–95)
6	26 (10–50)	57 (34–77)	69 (40–89)	57 (39–74)	84 (73–91)	90 (81–95)	89 (77–96)	98 (96–99)	88 (73–96)
7	18 (5–41)	49 (24–73)	63 (31–88)	49 (30–70)	82 (70–92)	90 (79–96)	89 (74–97)	99 (97–100)	87 (69–97)
8	12 (2–32)	42 (17–69)	58 (22–88)	42 (22–65)	81 (65–92)	89 (77–96)	88 (71–97)	99 (97–100)	86 (64–97)
9	8 (1–24)	35 (11–65)	53 (16–88)	35 (15–60)	79 (61–92)	89 (75–97)	88 (68–97)	99 (98–100)	86 (59–97)
10	5 (1–18)	29 (7–61)	48 (11–87)	29 (10–55)	77 (56–92)	89 (72–97)	87 (64–98)	99 (98–100)	85 (54–98)
11	3 (0–13)	24 (5–57)	44 (7–87)	23 (7–50)	76 (51–92)	89 (69–98)	87 (60–98)	100 (98–100)	84 (49–98)
12	2 (0–9)	19 (3–53)	40 (5–86)	18 (5–45)	74 (46–92)	88 (67–98)	86 (56–98)	100 (99–100)	83 (44–98)
13	1 (0–6)	16 (2–48)	36 (3–86)	15 (3–40)	72 (42–92)	88 (64–98)	86 (52–99)	100 (99–100)	82 (40–99)

CrI: credible interval; LFA: lateral flow assay; N: nucleocapsid; Q: quantitative; RBD: receptor-binding domain; S: spike; SARS-CoV-2: severe acute respiratory syndrome coronavirus 2.

Each row corresponds to a different type of test, with characteristics indicated in the first three columns. These estimates do not include the between-assay or between-study variability in their CrI. These sensitivities correspond to the red lines and shaded regions in Figure 3.

On the antigen effect, assays targeting the nucleocapsid showed faster seroreversion than those targeting the spike protein (*β_Nucleocapsid_ < β_Spike_* in 99.7% of the posterior samples). Assays targeting the RBD had on average slower seroreversion than those targeting the spike protein, although the effect was not statistically significant (*β_Spike_ < β_RBD_* in 87.3% of the samples). Differences between antigens can be appreciated by comparing the different rows of Figure 3.

To see how the different slopes translate to differences in sensitivity, the reader can compare the sensitivities of the different types of assays for a given delay between diagnosis and test in Table 1. Note that there was considerable variability between different assays of the same type (i.e. between the black lines within a same panel). We provide assay-specific sensitivity profiles in Supplementary Table S2. When estimating the extent of seroreversion for a given survey, assay-specific sensitivity estimates should be preferred over the coarser estimates provided for assay types.

All these results were robust to fitting separately a model using only assay antigen (the detailed results of this model fit are provided in Supplementary Figure S3) and a model using only analytic technique (detailed results provided in Supplementary Figure S4), and to excluding data points with estimated times from the model fit (these analyses are appended as extra material in Supplementary Figures S5 and S6). The full model had cross-validation accuracy similar to the original model, with data points falling in the 95% CrI of their predictions 92.0% of the time, and CrI were narrower (more precise) in 81% of the data points.

Finally, we tested whether specificity was also related to assay characteristics. Since specificity does not have temporal dynamics, we only analysed point estimates (see the details of the model in Supplement section G). Similar to sensitivity, we found that LFA assays have on average smaller specificities than quantitative assays (*β_LFA_ <* 0 in 98.4% of the posterior samples). Unlike sensitivity, we did not find significant differences with quantitative assays (e.g. *β_Direct_ >* 0 in 85.0% of the posterior samples) or between antigens (e.g. *β_RBD_ > β_N_* in 67.2% of the posterior samples). Differences in specificity between types of assays were of epidemiologically relevant magnitude (e.g. average specificity of 99.9% (95% CrI: 99.7–100) for RBD/quantitative-indirect assays and 98.8% (95% CrI: 96.6–99.7) for nucleocapsid/LFA assays; the authors make all estimates available in Supplementary Table S4). Like for sensitivity, we found considerable variability between studies reporting on the same assay (*σ_us_* = 0.61, 95% CrI: 0.20–1.14). Specificity data and the resulting fit are appended to this article in Supplementary Figure S7.

### Manufacturer sensitivities and risk of bias in the literature

Although quantitatively estimating and correcting the seroreversion bias in the literature is outside the scope of the present work, we can coarsely estimate the risk of seroreversion across the literature.

We compared our estimates to assay sensitivities provided by manufacturers, which report the percentage of serological samples from individuals diagnosed with COVID-19 that show a positive test result (if manufacturer values were missing, we used values reported by the United States Food and Drug Administration (FDA) or reported by authors). We found that 4 months after diagnosis, 20% of the assays have sensitivities below 75% of the originally specified value. At 6 months after diagnosis, 34% of the assays were below 75%. Thus, a few months after a COVID-19 wave, some serological assays (mostly LFA and quantitative-indirect assays targeting nucleocapsid antibodies) can severely underestimate previous infections.

We further analysed what percentage of serosurveys reported in the literature were at high risk of bias by seroreversion. As a reference, we used a comprehensive meta-analysis of the global evolution of SARS-CoV-2 seroprevalence [17], using the publicly available SeroTracker dataset [33], which notes the lack of seroreversion adjustment as a limitation. We estimated what percentage of the data points listed in SeroTracker, aligned with the World Health Organization (WHO) Unity protocol (i.e. those studies used in [17]), used assays with high rates of seroreversion (LFA assays or nucleocapsid quantitative-indirect assays). Because seroreversion depends on the assay used and on the time elapsed between an epidemic wave and serosurvey, we segregated the data across semesters.

We see in Table 2 that although the use of serological assays at high risk of seroreversion decreased throughout the pandemic, they still constituted a considerable fraction of Unity-aligned data points until mid-2021.

**Table 2:** Unity-aligned seroprevalence data points of the Serotracker dataset [33] that use assays at high risk of seroreversion, defined as lateral flow assays or quantitative-indirect assays for SARS-CoV-2 nucleocapsid antibodies, January 2020–December 2021 (n = 1,592)

Period of serological sampling	SeroTracker data points at high seroreversion risk (total data points)	Percentage of assays at high seroreversion risk (%)
1 Jan–30 Jun 2020	135 (506)	26.9
1 Jul–31 Dec 2020	140 (596)	23.4
1 Jan–30 Jun 2021	58 (330)	17.5
1 Jul–31 Dec 2021	10 (160)	6.3

SARS-CoV-2: severe acute respiratory syndrome coronavirus 2.

## Discussion

Serology-based estimates of infections are important to understand COVID-19. Although it is known that accounting for seroreversion in these estimates is important, there is a lack of appropriate data and guidelines to do so. Few studies correct for seroreversion [1,15,16,27,34,35], and the lack of robust assay-specific seroreversion estimates make it uncertain how accurate existing adjustments are. We present the first large-scale systematic analysis of seroreversion across dozens of seroassays for SARS-CoV-2, making three major contributions to help understand and correct for seroreversion.

Firstly, we provide time-varying sensitivity estimates for 50 assays and estimates of the average time-varying sensitivity for different assay types. These estimates can be used to adjust for seroreversion in the literature. Knowing the assay’s identity (or its characteristics, for assays not represented in our sample), and the time span between the epidemic wave and the serosurvey date at the tested location (which can be estimated from case or death curves), a seroreversion-adjusted sensitivity estimate can be selected from our results. Using these sensitivity estimates in the standard Rogan–Gladen formula will produce seroprevalence estimates that are corrected for seroreversion. Importantly, this procedure showed good performance at predicting assay sensitivity in a rigorous cross-validation analysis.

Our second contribution is the quantification of how seroreversion depends on assay characteristics. We show that seroreversion depends heavily on the antigen and on the analytical technology. Assays that use LFA technique (qualitative, rapid tests) show faster sensitivity decay, while quantitative assays with direct antibody binding have the slowest decay. This is in line with the high sensitivity of direct binding assays reported for other pathogens, ascribed to factors such as less sample diluting or the detection method not being limited to one class of antibodies [36,37]. Assays for nucleocapsid-targeting antibodies tended to decay faster than assays for spike protein antibodies, while assays targeting S1-RBD antibodies tended to decay more slowly (although this last effect was not significant at the 95% level). Interestingly, we found that one type of assay, the quantitative-direct assays targeting RBD-binding antibodies had on average increasing sensitivity over time. This is in line with previous studies reporting assays of this type to have increasing sensitivity, attributing this effect to prolonged antibody maturation [13,14]. Because reinfection incidence was likely to be low in our data, it is unlikely that these results reflect infections.

The striking differences between types of assays (e.g. average sensitivity at 6 months of 98% for S1-RBD-targeting quantitative-direct, against 26% for nucleocapsid LFA assays) outlines the need for assay-specific corrections. For example, the one-size-fits-all seroreversion rates (i.e. not assay-specific) used in two previous analyses of SARS-CoV-2 infection fatality rate (5% monthly decrease [35] and 190 days half-life [1]) would either considerably overestimate or underestimate seroreversion for many assays, according to our results. These results are in line with previous reports in the literature [13-15,20], although previous studies analysed fewer characteristics in general and did not quantify their effects. Our analysis also showed that specificity depends on assay characteristics.

These results will allow researchers to assess the risk of seroreversion bias in serosurveys, providing a valuable tool for the design of serological studies. For example, our results suggest that the strategy of comparing S1-RBD and nucleocapsid antibody prevalences to distinguish vaccine- and infection-induced population immunity can be affected by the different seroreversion rates of these assays [10,17,38].

Our third contribution is showing that a few months after diagnosis, manufacturer specifications can be unreliable for a considerable fraction of assays. Relatedly, we show that a sizable fraction of Unity-aligned serosurveys used in recent WHO estimates of global seroprevalence dynamics are at risk of seroreversion bias [17]. This underscores the potential of decaying sensitivity to bias our epidemiological understanding of COVID-19, and a potential interest of public health policymakers in ensuring that assay manufacturers and regulatory bodies provide information and guidelines regarding seroreversion [39]. The sensitivity estimates presented here should provide a straightforward way to correct for seroreversion in such datasets and to quantify literature bias.

To our knowledge, this is the most comprehensive analysis, for any pathogen, of assay-specific serological sensitivity decay and its dependence on assay characteristics. This is because some characteristics of the COVID-19 pandemic have allowed for a richer seroreversion dataset than is probably possible for any other pathogen (i.e. well approximated infection-to-testing times, multiple seroassays, multiple studies per seroassay, first exposures to a novel pathogen). Thus, many of the conclusions extracted from this analysis may serve as a guide for other emerging and endemic pathogens.

Our study has some limitations. Firstly, although we included more assays than previous studies, many of the included assays had seroreversion data for only a few time points. Secondly, we were unable to test the effects of important parameters such as age or disease severity on seroreversion [13,14,25,40]. Relatedly, although an ideal dataset would use a well defined cohort representative of the general population, with known age, sex ratio, disease severity, infecting variant and occurrence of reinfections, the available literature falls short of this ideal. This has the potential to introduce variability and biases in our estimates. We note, however, that our modelling framework is flexible, and could be extended to account for these variables, given appropriate data. Thirdly, as we analysed test data conditional on individuals having a previous COVID-19 diagnosis, it is likely that asymptomatic individuals were underrepresented in our sample. Finally, because our analysis included only data points on non-vaccinated individuals, and most of the included data points were sampled in 2020 when SARS-CoV-2 variants of concern and reinfections were uncommon, it is unclear how our results would extrapolate to antibodies induced by vaccines, reinfections or new variants of the virus.

## Conclusion

Accounting for seroreversion in serology-based estimates of infection numbers is important for understanding the COVID-19 pandemic, and for the usefulness of continued serological testing to monitor the effects of COVID-19. Rapid LFA tests as well as quantitative-indirect tests for nucleocapsid targeting antibodies have a high potential for seroreversion, and quantitative-direct assays are likely to be preferred for long-term serological surveillance. A considerable number of studies in the literature use assays with high risk of seroreversion, indicating some important potential for bias. We present a simple method for researchers to account for seroreversion when analysing serological data and when designing serological studies. This may be of interest to the management of other pathogens, and serosurveillance more in general, because of the unique opportunity to study the effects of seroreversion provided by the data generated during the COVID-19 pandemic.

## Supplementary Data

3457000 Supplement
