# Supplementary material for: Dynamics of SARS-CoV-2 seroassay sensitivity: a systematic review and modelling study
Source: Euro Surveill. 2023 May 25;28(21):2200809. doi: 10.2807/1560-7917.ES.2023.28.21.2200809 (PMC10283460; doi:10.2807/1560-7917.ES.2023.28.21.2200809)
Supplement: Supplement [file 22-00809_HERRERA-ESPOSITO_Supplement.pdf]

Supplementary material:

This supplementary material is hosted by *Eurosurveillance* as supporting information alongside the article "Dynamics of SARS-CoV-2 seroassay sensitivity: a systematic review and modeling study", on behalf of the authors, who remain responsible for the accuracy and appropriateness of the content. The same standards for ethics, copyright, attributions and permissions as for the article apply. Supplements are not edited by *Eurosurveillance* and the journal is not responsible for the maintenance of any links or email addresses provided therein.

Dynamics of SARS-CoV-2 seroassay sensitivity: a systematic review and modeling study

Index:

A) **Supplementary methods, literature review……………………………….. p.2**
B) **Supplementary methods, statistical modelling ………………………… p.24**
C) **Assay-specific sensitivity profiles ……………………………..…….. p.26**
D) **Assay sensitivity increase can continue across months ……..………. p.37**
E) **Robustness to model architecture ..………………………………………. p.38**
F) **Robustness to time estimation ..…………………………………………… p.41**
G) **Effect of assay characteristics on specificity…………………………….. p.43**

A) Supplementary methods, literature review

Inclusion and exclusion criteria of the systematic review

We set the following list of inclusion requirements for every study:

- Serologically tested unvaccinated individuals at least one month after a non-serological COVID-19 diagnosis
- Specified a median month of sampling, or both a sampling start-month and end-month
- Reported either confidence intervals for sensitivity, or both the number of people tested and the number of positive tests
- Used at least one commercial assay, or an assay used for seroprevalence studies (e.g. excluding studies reporting on in-house assays used only for clinical testing)

We set the following list of exclusion criteria:

- The tested population was a subset of the tested population from another included study
- Only reported the results of multiple simultaneously used assays instead of reporting assay results individually.
- Only tested sample frames likely to exclude particular age groups and other demographics (e.g. nursing home residents, transplant recipients, blood donors, children, healthcare workers, and workplace cohorts that excluded children; see (1) for further discussion)
- Only tested hospitalised patients (see (2) for further discussion)
- Used only the Wondfo or Menarini assays, which have been reported to have large between-batch variability (3,4)
- Were performed in Russia and had unknown times between diagnosis and serological sampling, since Russia's case reporting does not allow for reliable estimate of these times (see (5) for an analysis of Russia's case reporting).

The detailed list of studies and review results are available at the associated GitHub page. The review and review protocol were not registered.

Assay characteristics table

In **Table S1** we show the list of assays included in the study, indicating the main characteristics of each one, as well as the sources from which the data of each assay was obtained.

Table S1. List of included assays, together with data sources and assay characteristics. Note that we only indicate the “best” antigen for each assay. That is, if an assay detects antibodies targeting the nucleocapsid and the spike protein, it will appear as “Spike” in the table.

Assay	References	Antigen	Design
Abbott Alinity	(6,7)	Nucleocapsid	Quantitative-Indirect
Abbott Alinity II Quant	(8)	Receptor-binding domain	Quantitative-Indirect
Abbott Architect	(2,6,8–26)	Nucleocapsid	Quantitative-Indirect
AMP rapid test	(27)	Unknown	LFA
Abingdon AbC-19	(4)	Spike	LFA
Beckman ACCESS	(28)	Receptor-binding domain	Quantitative-Indirect
Biomerica rapid test	(4)	Nucleocapsid	LFA
BioRad Platelia	(29)	Nucleocapsid	Quantitative-Direct
Biozek rapid test	(4)	Receptor-binding domain	LFA
CCAHS	(30)	Receptor-binding domain	Quantitative-Indirect
COVIDAR	(31–33)	Receptor-binding domain	Quantitative-Indirect
Diasorin Liaison	(7,9–11,22,24,25,34)	Spike	Quantitative-Indirect
EDI Novel Coronavirus	(35)	Nucleocapsid	Quantitative-Indirect
Elecsys anti-N	(2,6,7,9–11,24,34–44)	Nucleocapsid	Quantitative-Direct
Elecsys anti-S	(10)	Receptor-binding domain	Quantitative-Direct
ERBALISA	(45,46)	Spike	Quantitative-Indirect
Euroimmun anti-S	(2,7,10,12,22,23,25,35,47–58)	Spike	Quantitative-Indirect
Euroimmun anti-N	(10,23)	Nucleocapsid	Quantitative-Indirect
Fortress Diagnostics	(59–61)	Spike	LFA
GenScript cPass	(8,10,23)	Receptor-binding domain	Quantitative-Competitive
Hangzhou Clongene	(62)	Unknown	LFA
IDK (Immundiagnostik)	(7)	Spike	Quantitative-Indirect
InBios	(35)	Spike	Quantitative-Indirect
Mehdi, in-house	(21)	Spike	Quantitative-Indirect
THL Finland, in-house FMIA	(63)	Spike	Quantitative-Indirect
THL Finland, in-house neutralisation assay	(63–65)	Receptor-binding domain	Quantitative-Competitive

Gauteng, in-house Luminex	(66)	Receptor-binding domain	Quantitative-Unknown
Tunis, in-house	(67)	Receptor-binding domain	Quantitative-Unknown
Kavach ELISA IgG (Zydus)	(45)	Receptor-binding domain	Quantitative-Indirect
Livzon lateral flow	(68,69)	Unknown	LFA
LumiraDx	(4)	Receptor-binding domain	Quantitative-Unknown
Luminex xMAP (RBD portion)	(70)	Receptor-binding domain	Quantitative-Indirect
Massachusetts General Hospital	(71)	Receptor-binding domain	Quantitative-Indirect
Mount Sinai (New York)	(72)	Receptor-binding domain	Quantitative-Indirect
Roche rapid test	(4)	Spike	LFA
Orient Gene Biotech	(13,73,74)	Spike	LFA
SenASTrIS test	(75,76)	Spike	Quantitative-Indirect
Siemens Advia Centaur	(24,77)	Receptor-binding domain	Quantitative-Direct
Siemens Advia anti-RBD	(20)	Receptor-binding domain	Quantitative-Indirect
Standard Q	(78)	Receptor-binding domain	LFA
Sinai Health (Toronto)	(79)	Spike	Quantitative-Indirect
Techno Genetics	(80)	Receptor-binding domain	LFA
Tosoh N sandwich	(43)	Nucleocapsid	Quantitative-Direct
Tosoh N indirect	(43)	Nucleocapsid	Quantitative-Indirect
Tosoh S sandwich	(43)	Receptor-binding domain	Quantitative-Direct
Tosoh S indirect	(43)	Receptor-binding domain	Quantitative-Indirect
University of Rio de Janeiro	(3,53)	Spike	Quantitative-Indirect
Vitros Ortho (not total antibodies)	(9,81–84)	Spike	Quantitative-Indirect
Vitros Ortho (total antibodies)	(9,10)	Receptor-binding domain	Quantitative-Direct
Wantai	(10,42,85–88)	Receptor-binding domain	Quantitative-Direct

Data extraction and risk of bias assessment

Two authors reviewed the studies, screened for inclusion, extracted data, and assessed studies for risk of bias. All disagreements were solved by consensus via email and bi-monthly

meetings. Where essential data were missing, we requested the data from study authors by email.

We extracted the following data for each included study:
- Nation or subnational location from which the sample was drawn
- Sample frame
- Median month of sampling, and/or both a sampling start-month and end-month
- Either confidence intervals for sensitivity, and/or both the number of people tested and the number of positive tests
- Name of the serological assay
- Reported median (or mean) age and sex balance of the cohort
- Reported severity of infection in the cohort

For each included assay we sought reported sensitivity from the manufacturer or other published source.

We assessed studies as low risk of bias if participants were drawn from a random sample taken in a sample frame representative of the general population, or from sampling the majority of the population by census. Detailed assessment comments are available in the associated GitHub page (Methods section, main text). Studies were assessed as high risk when participants were drawn from an alternative sample, such as a convenience sample of non-hospitalized individuals. Publication bias is unlikely to strongly skew our results because studies with high or low seropositivity rates are of scientific interest; prior work found no evidence of publication bias in seroprevalence studies within wealthier nations (85). We sought to further minimise risk of publication bias by extensively searching the grey literature and following up on media reports (86).

In **Table S2**, we show a list of the included studies and each data point included in our analysis, indicating different relevant cohort characteristics. We observe that the median age of the studies falls mostly in the range around 40 years, as one would expect from representative samples of the adult population, with some somewhat older and some younger cohorts. The sex ratio in the samples is also mostly centred around 50% female participants, with some studies reporting somewhat unbalanced samples (e.g. 70% female, 30% male), suggesting that some studies depart from the expected representative ratios (although some cohorts are small, and random variability in sex ratio can be expected to be large). We also show the infection severity of the cohorts when these are reported. The majority of studies reporting the severity of the cohort have severity ranges under 10%, which

is in the range expected from the rate of severe disease among diagnosed individuals in 2020 (89), when most studies were performed. Some studies report higher hospitalisation rates, indicating that these samples are not representative of the general population. It is likely that most of the studies not reporting on the rate of severe disease include only small numbers of patients with severe disease (i.e. they are not sampling a cohort where hospitalised patients are overrepresented and where reporting on severity would be crucial). Under this assumption, most of included studies have rates of severe disease within the expected range. In sum, the reported ages, sex ratios and severity rates of analyzed cohorts indicate that most data points are broadly in line with the expectations of a representative population sampling. Some cohorts depart from the expected representative numbers, indicating a potential to bias the obtained results. However, because most data points have apparently representative cohorts, and non-representative cohorts are distributed among the different types of tests, we expect the potential for bias from cohort characteristics to be small.

Two further concerns regarding potential bias in the results are the effects of infection by different variants, as well as the potential for reinfections. No studies report on the infecting variant of the tested individuals, and only one study reports on the absence of reinfections (22). However, both the emergence of new SARS-CoV-2 variants and reinfections were not common during most of 2020, and so we assess that the serology samples collected in 2020 are likely due to infections from the original variant, and at low risk of reinfections. We see in **Table S2** that most analysed samples (90%, 217/242 data points with reported sampling date) were collected in 2020, suggesting that our results are mostly representative of the original variant, and at low risk of bias due to reinfections.

Table S2. Characteristics of serological studies and analysed cohorts for each data point included in the study. Empty cells indicate that the value is not reported in the study. For rows with empty time between diagnosis and serology, we estimated the times as reported in the Methods section in the main text. Each row corresponds to one data point in **Figure S1** below.

Reference	Serology sampling start date	Serology sampling end date	Serology sampling midpoint	Time between prior diagnosis (or symptoms) and serology (Months)	Assay	Severity of infection	Median age	Sex ratio	Notes
2	NA	October 2020	October 2020	6	Abbott Architect	0% hospitalized	NA	NA	
2	NA	October 2020	October 2020	6	Elecsys anti-N	0% hospitalized	NA	NA	
2	NA	October 2020	October 2020	6	Euroimmun anti-S	0% hospitalized	NA	NA	
3	NA	NA	November 2020	1	University of Rio de Janeiro	NA	30 - 50	58% female , 42% male	table 2
3	NA	NA	November 2020	2	University of Rio de Janeiro	NA	30 - 50	58% female , 42% male	table 2
3	NA	NA	November 2020	3	University of Rio de Janeiro	NA	30 - 50	58% female , 42% male	table 2
3	NA	NA	November 2020	4	University of Rio de Janeiro	NA	30 - 50	58% female , 42% male	table 2
3	NA	NA	November 2020	5	University of Rio de Janeiro	NA	30 - 50	58% female , 42% male	table 2
4	September 2020	November 2020	NA	2	Abingdon AbC-19	2.6% hospitalized	NA	NA	
4	September 2020	November 2020	NA	4	Abingdon AbC-19	2.6% hospitalized	NA	NA	
4	September 2020	November 2020	NA	6	Abingdon AbC-19	2.6% hospitalized	NA	NA	
4	September 2020	November 2020	NA	1	Biomerica rapid test	2.6% hospitalized	NA	NA	
4	September 2020	November 2020	NA	2	Biomerica rapid test	2.6% hospitalized	NA	NA	

4	September 2020	November 2020	NA	4	Biomerica rapid test	2.6% hospitalized	NA	NA	
4	September 2020	November 2020	NA	6	Biomerica rapid test	2.6% hospitalized	NA	NA	
4	September 2020	November 2020	NA	1	Biozek rapid test	2.6% hospitalized	NA	NA	
4	September 2020	November 2020	NA	2	Biozek rapid test	2.6% hospitalized	NA	NA	
4	September 2020	November 2020	NA	1	LumiraDx	2.6% hospitalized	NA	NA	
4	September 2020	November 2020	NA	2	LumiraDx	2.6% hospitalized	NA	NA	
4	September 2020	November 2020	NA	4	LumiraDx	2.6% hospitalized	NA	NA	
4	September 2020	November 2020	NA	6	LumiraDx	2.6% hospitalized	NA	NA	
4	September 2020	November 2020	NA	1	Roche rapid test	2.6% hospitalized	NA	NA	
4	September 2020	November 2020	NA	2	Roche rapid test	2.6% hospitalized	NA	NA	
4	September 2020	November 2020	NA	4	Roche rapid test	2.6% hospitalized	NA	NA	
4	September 2020	November 2020	NA	6	Roche rapid test	2.6% hospitalized	NA	NA	
6	3/26/2020	7/10/2020	5/18/2020	1	Abbott Alinity	72% hospitalized	57	46% female , 54% male	table 1
6	3/26/2020	7/10/2020	5/18/2020	1	Abbott Architect	72% hospitalized	57	46% female , 54% male	table 1
6	3/26/2020	7/10/2020	5/18/2020	1	Elecsys anti-N	72% hospitalized	57	46% female , 54% male	table 1
7	August 2020	September 2020	NA	5	Abbott Alinity	4% severe	44	55% female , 45% male	table 2

7	August 2020	September 2020	NA	5	Diasorin Liaison	4% severe	44	55% female , 45% male	table 2
7	August 2020	September 2020	NA	5	Elecsys anti-N	4% severe	44	55% female , 45% male	table 2
7	August 2020	September 2020	NA	5	Euroimmun anti-S	4% severe	44	55% female , 45% male	table 2
7	August 2020	September 2020	NA	5	IDK (Immundiagnostik)	4% severe	44	55% female , 45% male	table 2
8	3/11/2020	4/5/2020	NA	10	Abbott Alinity II Quant	NA	52	70% female , 30% male	table 1
8	3/11/2020	4/5/2020	NA	10	Abbott Architect	NA	52	70% female , 30% male	table 1
8	3/11/2020	4/5/2020	NA	10	GenScript cPass	NA	52	70% female , 30% male	table 1
9	April 2020	July 2020	NA	1	Abbott Architect	0% hospitalized	30 - 45	48% female , 52% male	supplementary table 1
9	NA	NA	NA	3	Abbott Architect	0% hospitalized	30 - 45	48% female , 52% male	supplementary table 1
9	NA	NA	NA	5	Abbott Architect	0% hospitalized	30 - 45	48% female , 52% male	supplementary table 1
9	NA	NA	NA	7	Abbott Architect	0% hospitalized	30 - 45	48% female , 52% male	supplementary table 1
9	April 2020	July 2020	NA	1	Diasorin Liaison	0% hospitalized	30 - 45	48% female , 52% male	supplementary table 1
9	NA	NA	NA	3	Diasorin Liaison	0% hospitalized	30 - 45	48% female , 52% male	supplementary table 1
9	NA	NA	NA	5	Diasorin Liaison	0% hospitalized	30 - 45	48% female , 52% male	supplementary table 1

9	NA	NA	NA	7	Diasorin Liaison	0% hospitalized	30 - 45	48% female , 52% male	supplementary table 1
9	April 2020	July 2020	NA	1	Elecsys anti-N	0% hospitalized	30 - 45	48% female , 52% male	supplementary table 1
9	NA	NA	NA	3	Elecsys anti-N	0% hospitalized	30 - 45	48% female , 52% male	supplementary table 1
9	NA	NA	NA	5	Elecsys anti-N	0% hospitalized	30 - 45	48% female , 52% male	supplementary table 1
9	NA	NA	NA	7	Elecsys anti-N	0% hospitalized	30 - 45	48% female , 52% male	supplementary table 1
9	April 2020	July 2020	NA	1	Vitros Ortho (not total antibodies)	0% hospitalized	30 - 45	48% female , 52% male	supplementary table 1
9	NA	NA	NA	3	Vitros Ortho (not total antibodies)	0% hospitalized	30 - 45	48% female , 52% male	supplementary table 1
9	NA	NA	NA	5	Vitros Ortho (not total antibodies)	0% hospitalized	30 - 45	48% female , 52% male	supplementary table 1
9	NA	NA	NA	7	Vitros Ortho (not total antibodies)	0% hospitalized	30 - 45	48% female , 52% male	supplementary table 1
9	April 2020	July 2020	NA	1	Vitros Ortho (total antibodies)	0% hospitalized	30 - 45	48% female , 52% male	supplementary table 1
9	NA	NA	NA	3	Vitros Ortho (total antibodies)	0% hospitalized	30 - 45	48% female , 52% male	supplementary table 1
9	NA	NA	NA	5	Vitros Ortho (total antibodies)	0% hospitalized	30 - 45	48% female , 52% male	supplementary table 1
9	NA	NA	NA	7	Vitros Ortho (total antibodies)	0% hospitalized	30 - 45	48% female , 52% male	supplementary table 1
10	NA	NA	2020	1	Abbott Architect	15.8% hospitalized	NA	NA	
10	NA	NA	2020	2	Abbott Architect	15.8% hospitalized	NA	NA	
10	NA	NA	2020	4	Abbott Architect	15.8% hospitalized	NA	NA	

10	NA	NA	2020	6	Abbott Architect	15.8% hospitalized	NA	NA	
10	NA	NA	2020	8	Abbott Architect	15.8% hospitalized	NA	NA	
10	NA	NA	2020	10	Abbott Architect	15.8% hospitalized	NA	NA	
10	NA	NA	2020	12	Abbott Architect	15.8% hospitalized	NA	NA	
10	NA	NA	2020	14	Abbott Architect	15.8% hospitalized	NA	NA	
10	NA	NA	2020	1	Diasorin Liaison	15.8% hospitalized	NA	NA	
10	NA	NA	2020	2	Diasorin Liaison	15.8% hospitalized	NA	NA	
10	NA	NA	2020	4	Diasorin Liaison	15.8% hospitalized	NA	NA	
10	NA	NA	2020	6	Diasorin Liaison	15.8% hospitalized	NA	NA	
10	NA	NA	2020	8	Diasorin Liaison	15.8% hospitalized	NA	NA	
10	NA	NA	2020	10	Diasorin Liaison	15.8% hospitalized	NA	NA	
10	NA	NA	2020	12	Diasorin Liaison	15.8% hospitalized	NA	NA	
10	NA	NA	2020	14	Diasorin Liaison	15.8% hospitalized	NA	NA	
10	NA	NA	2020	1	Elecsys anti-N	15.8% hospitalized	NA	NA	
10	NA	NA	2020	2	Elecsys anti-N	15.8% hospitalized	NA	NA	
10	NA	NA	2020	4	Elecsys anti-N	15.8% hospitalized	NA	NA	

10	NA	NA	2020	6	Elecsys anti-N	15.8% hospitalized	NA	NA	
10	NA	NA	2020	8	Elecsys anti-N	15.8% hospitalized	NA	NA	
10	NA	NA	2020	10	Elecsys anti-N	15.8% hospitalized	NA	NA	
10	NA	NA	2020	12	Elecsys anti-N	15.8% hospitalized	NA	NA	
10	NA	NA	2020	14	Elecsys anti-N	15.8% hospitalized	NA	NA	
10	NA	NA	2020	1	Elecsys anti-S	15.8% hospitalized	NA	NA	
10	NA	NA	2020	2	Elecsys anti-S	15.8% hospitalized	NA	NA	
10	NA	NA	2020	4	Elecsys anti-S	15.8% hospitalized	NA	NA	
10	NA	NA	2020	6	Elecsys anti-S	15.8% hospitalized	NA	NA	
10	NA	NA	2020	8	Elecsys anti-S	15.8% hospitalized	NA	NA	
10	NA	NA	2020	10	Elecsys anti-S	15.8% hospitalized	NA	NA	
10	NA	NA	2020	12	Elecsys anti-S	15.8% hospitalized	NA	NA	
10	NA	NA	2020	14	Elecsys anti-S	15.8% hospitalized	NA	NA	
10	NA	NA	2020	1	Euroimmun anti-N	15.8% hospitalized	NA	NA	
10	NA	NA	2020	2	Euroimmun anti-N	15.8% hospitalized	NA	NA	
10	NA	NA	2020	4	Euroimmun anti-N	15.8% hospitalized	NA	NA	

10	NA	NA	2020	6	Euroimmun anti-N	15.8% hospitalized	NA	NA	
10	NA	NA	2020	8	Euroimmun anti-N	15.8% hospitalized	NA	NA	
10	NA	NA	2020	10	Euroimmun anti-N	15.8% hospitalized	NA	NA	
10	NA	NA	2020	12	Euroimmun anti-N	15.8% hospitalized	NA	NA	
10	NA	NA	2020	14	Euroimmun anti-N	15.8% hospitalized	NA	NA	
10	NA	NA	2020	1	Euroimmun anti-S	15.8% hospitalized	NA	NA	
10	NA	NA	2020	2	Euroimmun anti-S	15.8% hospitalized	NA	NA	
10	NA	NA	2020	4	Euroimmun anti-S	15.8% hospitalized	NA	NA	
10	NA	NA	2020	6	Euroimmun anti-S	15.8% hospitalized	NA	NA	
10	NA	NA	2020	8	Euroimmun anti-S	15.8% hospitalized	NA	NA	
10	NA	NA	2020	10	Euroimmun anti-S	15.8% hospitalized	NA	NA	
10	NA	NA	2020	12	Euroimmun anti-S	15.8% hospitalized	NA	NA	
10	NA	NA	2020	14	Euroimmun anti-S	15.8% hospitalized	NA	NA	
10	NA	NA	2020	1	GenScript cPass	15.8% hospitalized	NA	NA	
10	NA	NA	2020	2	GenScript cPass	15.8% hospitalized	NA	NA	
10	NA	NA	2020	4	GenScript cPass	15.8% hospitalized	NA	NA	

10	NA	NA	2020	6	GenScript cPass	15.8% hospitalized	NA	NA	
10	NA	NA	2020	8	GenScript cPass	15.8% hospitalized	NA	NA	
10	NA	NA	2020	10	GenScript cPass	15.8% hospitalized	NA	NA	
10	NA	NA	2020	12	GenScript cPass	15.8% hospitalized	NA	NA	
10	NA	NA	2020	14	GenScript cPass	15.8% hospitalized	NA	NA	
10	NA	NA	2020	1	Vitros Ortho (total antibodies)	15.8% hospitalized	NA	NA	
10	NA	NA	2020	2	Vitros Ortho (total antibodies)	15.8% hospitalized	NA	NA	
10	NA	NA	2020	4	Vitros Ortho (total antibodies)	15.8% hospitalized	NA	NA	
10	NA	NA	2020	6	Vitros Ortho (total antibodies)	15.8% hospitalized	NA	NA	
10	NA	NA	2020	8	Vitros Ortho (total antibodies)	15.8% hospitalized	NA	NA	
10	NA	NA	2020	10	Vitros Ortho (total antibodies)	15.8% hospitalized	NA	NA	
10	NA	NA	2020	12	Vitros Ortho (total antibodies)	15.8% hospitalized	NA	NA	
10	NA	NA	2020	14	Vitros Ortho (total antibodies)	15.8% hospitalized	NA	NA	
10	NA	NA	2020	1	Wantai	15.8% hospitalized	NA	NA	
10	NA	NA	2020	2	Wantai	15.8% hospitalized	NA	NA	
10	NA	NA	2020	4	Wantai	15.8% hospitalized	NA	NA	

10	NA	NA	2020	6	Wantai	15.8% hospitalized	NA	NA	
10	NA	NA	2020	8	Wantai	15.8% hospitalized	NA	NA	
10	NA	NA	2020	10	Wantai	15.8% hospitalized	NA	NA	
10	NA	NA	2020	12	Wantai	15.8% hospitalized	NA	NA	
10	NA	NA	2020	14	Wantai	15.8% hospitalized	NA	NA	
11	NA	NA	NA	1	Abbott Architect	~26% hospitalized	40 - 45	~42% female , ~58% male	supplementary data #2
11	NA	NA	NA	1 - 3	Abbott Architect	~26% hospitalized	40 - 45	~42% female , ~58% male	supplementary data #2
11	NA	NA	NA	4 - 5	Abbott Architect	~26% hospitalized	40 - 45	~42% female , ~58% male	supplementary data #2
11	NA	NA	NA	5 - 7	Abbott Architect	~26% hospitalized	40 - 45	~42% female , ~58% male	supplementary data #2
11	NA	NA	NA	1	Diasorin Liaison	~26% hospitalized	40 - 45	~42% female , ~58% male	supplementary data #2
11	NA	NA	NA	1 - 3	Diasorin Liaison	~26% hospitalized	40 - 45	~42% female , ~58% male	supplementary data #2
11	NA	NA	NA	4 - 5	Diasorin Liaison	~26% hospitalized	40 - 45	~42% female , ~58% male	supplementary data #2
11	NA	NA	NA	5 - 7	Diasorin Liaison	~26% hospitalized	40 - 45	~42% female , ~58% male	supplementary data #2
11	NA	NA	NA	1	Elecsys anti-N	~26% hospitalized	40 - 45	~42% female , ~58% male	supplementary data #2
11	NA	NA	NA	1 - 3	Elecsys anti-N	~26% hospitalized	40 - 45	~42% female , ~58% male	supplementary data #2
11	NA	NA	NA	4 - 5	Elecsys anti-N	~26% hospitalized	40 - 45	~42% female , ~58% male	supplementary data #2

11	NA	NA	NA	5 - 7	Elecsys anti-N	~26% hospitalized	40 - 45	~42% female , ~58% male	supplementary data #2
12	4/21/2020	4/27/2020	4/24/2020	NA	Abbott Architect	< 9% hospitalized	30 - 50	~50%	supplementary figure 5B
12	4/21/2020	4/27/2020	4/24/2020	NA	Euroimmun anti-S	< 9% hospitalized	30 - 50	~50%	supplementary figure 5B
13	4/27/2020	5/11/2020	5/4/2020	NA	Abbott Architect	NA	NA	NA	
13	4/27/2020	5/11/2020	5/4/2020	NA	Orient Gene Biotech	NA	NA	NA	
14	5/1/2020	6/30/2020	5/31/2020	NA	Abbott Architect	NA	NA	NA	
15	6/24/2020	7/8/2020	7/1/2020	NA	Abbott Architect	NA	NA	NA	
16	8/17/2020	9/22/2020	9/4/2020	NA	Abbott Architect	NA	NA	NA	
17	10/17/2020	10/20/2020	10/18/2020	NA	Abbott Architect	NA	NA	NA	
18	10/17/2020	11/4/2020	10/26/2020	NA	Abbott Architect	~49% symptomatic	NA	NA	figure 3
19	6/14/2021	7/6/2021	6/25/2021	NA	Abbott Architect	NA	NA	NA	
20	NA	NA	12/27/2020	0.5 - 2	Abbott Architect	NA	NA	NA	
20	NA	NA	12/27/2020	2 - 4	Abbott Architect	NA	NA	NA	
20	NA	NA	12/27/2020	4 - 6	Abbott Architect	NA	NA	NA	
20	NA	NA	12/27/2020	>6	Abbott Architect	NA	NA	NA	
20	NA	NA	12/27/2020	0.5 - 2	Siemens Advia anti-RBD	NA	NA	NA	
20	NA	NA	12/27/2020	2 - 4	Siemens Advia anti-RBD	NA	NA	NA	
20	NA	NA	12/27/2020	4 - 6	Siemens Advia anti-RBD	NA	NA	NA	
20	NA	NA	12/27/2020	>6	Siemens Advia anti-RBD	NA	NA	NA	
21	NA	NA	12/18/2020	2	Abbott Architect	18% require oxygen	40	30% female , 70% male	table 1
21	NA	NA	12/18/2020	2	Mehdi, in-house	18% require oxygen	40	30% female , 70% male	table 1

22	March 2020	August 2020	NA	2	Abbott Architect	12% hospitalized	48	58% female , 42% male	table 1
22	March 2020	August 2020	NA	2	Diasorin Liaison	12% hospitalized	48	58% female , 42% male	table 1
22	March 2020	August 2020	NA	2	Euroimmun anti-S	12% hospitalized	48	58% female , 42% male	table 1
23	4/14/2020	NA	NA	2	Euroimmun anti-N	NA	NA	NA	
23	NA	8/15/2020	NA	4	Euroimmun anti-N	NA	NA	NA	
23	4/14/2020	NA	NA	2	Euroimmun anti-S	NA	NA	NA	
23	NA	8/15/2020	NA	4	Euroimmun anti-S	NA	NA	NA	
23	4/14/2020	NA	NA	2	GenScript cPass	NA	NA	NA	
23	NA	8/15/2020	NA	4	GenScript cPass	NA	NA	NA	
24	NA	NA	NA	3	Abbott Architect	0% hospitalized	44	72% female , 28% male	
24	NA	NA	NA	3	Diasorin Liaison	0% hospitalized	44	72% female , 28% male	
24	NA	NA	NA	3	Elecsys anti-N	0% hospitalized	44	72% female , 28% male	
24	NA	NA	NA	3	Siemens Advia Centaur	0% hospitalized	44	72% female , 28% male	
25	August 2020	October 2020	September 2020	5	Abbott Architect	7% hospitalized	46	52% female , 48% male	table 1
25	January 2021	March 2021	February 2021	10	Abbott Architect	7% hospitalized	46	52% female , 48% male	table 1
25	August 2020	October 2020	September 2020	5	Diasorin Liaison	7% hospitalized	46	52% female , 48% male	table 1
25	January 2021	March 2021	February 2021	10	Diasorin Liaison	7% hospitalized	46	52% female , 48% male	table 1
25	August 2020	October 2020	September 2020	5	Euroimmun anti-S	7% hospitalized	46	52% female , 48% male	table 1
25	January 2021	March 2021	February 2021	10	Euroimmun anti-S	7% hospitalized	46	52% female , 48% male	table 1

26	7/17/2020	10/15/2020	8/31/2020	1 - 2	Abbott Architect	NA	NA	NA	
26	7/17/2020	10/15/2020	8/31/2020	2 - 3	Abbott Architect	NA	NA	NA	
26	7/17/2020	10/15/2020	8/31/2020	3 - 4	Abbott Architect	NA	NA	NA	
26	7/17/2020	10/15/2020	8/31/2020	4 - 5	Abbott Architect	NA	NA	NA	
27	8/10/2020	9/10/2020	8/25/2020	NA	AMP rapid test	NA	NA	NA	email from study authors
28	8/15/2020	9/5/2020	8/25/2020	NA	Beckman ACCESS	0% hospitalized	NA	NA	
28	9/12/2020	10/24/2020	10/3/2020	NA	Beckman ACCESS	0% hospitalized	NA	NA	
28	11/7/2020	12/19/2020	11/28/2020	NA	Beckman ACCESS	0% hospitalized	NA	NA	
29	NA	NA	10/30/2020	NA	BioRad Platelia	NA	NA	NA	
30	11/2/2020	4/30/2021	1/30/2021	NA	CCAHS	NA	NA	NA	
31	03/03/2021	4/17/2021	03/25/2021	NA	COVIDAR	~4% hospitalized	39	61% female , 39% male	positive on nasal swab (supplemental S2 data)
32	NA	NA	NA	1	COVIDAR	NA	NA	NA	
33	NA	September 2020	NA	1	COVIDAR	10% severe	37 - 45	60% female , 40% male	table 1
33	NA	September 2020	NA	3	COVIDAR	12% severe	36 - 50	65% female , 35% male	table 1
34	5/1/2020	5/3/2020	5/2/2020	2	Abbott Architect	NA	NA	NA	
34	5/1/2020	5/3/2020	5/2/2020	2	Diasorin Liaison	NA	NA	NA	
34	5/1/2020	5/3/2020	5/2/2020	2	Elecsys anti-N	NA	NA	NA	
35	NA	NA	NA	8	EDI Novel Coronavirus	0% hospitalized	26	60% female , 40% male	tables 1 and 2
35	NA	NA	NA	8	Elecsys anti-N	0% hospitalized	26	60% female , 40% male	tables 1 and 2

35	NA	NA	NA	8	Euroimmun anti-S	0% hospitalized	26	60% female , 40% male	tables 1 and 2
35	NA	NA	NA	8	InBios	0% hospitalized	26	60% female , 40% male	tables 1 and 2
36	8/11/2020	8/16/2020	8/13/2020	NA	Elecsys anti-N	NA	NA	NA	
36	9/10/2020	9/16/2020	9/13/2020	NA	Elecsys anti-N	NA	NA	NA	
36	10/12/2020	10/16/2020	10/14/2020	NA	Elecsys anti-N	NA	NA	NA	
37	11/23/2020	2/19/2021	1/6/2021	NA	Elecsys anti-N	NA	NA	NA	
38	1/8/2021	1/24/2021	1/16/2021	NA	Elecsys anti-N	NA	NA	NA	
39	12/3/2021	12/27/2021	12/15/2021	NA	Elecsys anti-N	NA	NA	NA	
39	2/2/2022	3/6/2022	2/18/2022	NA	Elecsys anti-N	NA	NA	NA	
40	8/6/2020	8/31/2020	8/18/2020	1	Elecsys anti-N	NA	NA	NA	
41	NA	NA	8/26/2020	1	Elecsys anti-N	NA	NA	NA	
41	NA	NA	9/18/2020	1	Elecsys anti-N	NA	NA	NA	
42	4/3/2020	7/8/2020	5/21/2020	3	Elecsys anti-N	3% hospitalized	43	52% female , 48% male	table S1 in appendix 1, appendix 2
42	4/3/2020	7/8/2020	5/21/2020	3	Wantai	3% hospitalized	43	52% female , 48% male	table S1 in appendix 1, appendix 2
43	9/2/2020	10/26/2020	9/29/2020	6	Elecsys anti-N	73% hospitalized	50	50% female , 50% male	table 1
43	9/2/2020	10/26/2020	9/29/2020	6	Tosoh N indirect	73% hospitalized	50	50% female , 50% male	table 1
43	9/2/2020	10/26/2020	9/29/2020	6	Tosoh N sandwich	73% hospitalized	50	50% female , 50% male	table 1
43	9/2/2020	10/26/2020	9/29/2020	6	Tosoh S indirect	73% hospitalized	50	50% female , 50% male	table 1
43	9/2/2020	10/26/2020	9/29/2020	6	Tosoh S sandwich	73% hospitalized	50	50% female , 50% male	table 1

44	NA	late 2020	NA	9	Elecsys anti-N	3% hospitalized	57	19% female , 81% male	table 2
45	9/1/2020	9/7/2020	9/4/2020	NA	ERBALISA	NA	NA	NA	
45	10/15/2020	10/21/2020	10/18/2020	NA	ERBALISA	NA	NA	NA	
45	8/1/2020	8/7/2020	8/4/2020	NA	Kavach ELISA IgG (Zydus)	NA	NA	NA	
46	7/27/2020	8/7/2020	8/1/2020	2	ERBALISA	NA	NA	NA	
47	NA	NA	5/21/2020	NA	Euroimmun anti-S	NA	NA	NA	
48	5/4/2020	6/23/2020	5/29/2020	NA	Euroimmun anti-S	NA	NA	NA	
49	5/20/2020	6/9/2020	5/30/2020	NA	Euroimmun anti-S	34% moderate or severe	52	NA	table 3
50	7/20/2020	8/5/2020	7/28/2020	NA	Euroimmun anti-S	NA	NA	NA	
51	7/1/2020	7/28/2020	7/14/2020	NA	Euroimmun anti-S	NA	46 - 65	57% female, 43% male	supplement tables 1 + 2
51	8/5/2020	9/1/2020	8/18/2020	NA	Euroimmun anti-S	NA	46 - 65	57% female, 43% male	supplement tables 1 + 2
51	9/9/2020	10/9/2020	9/24/2020	NA	Euroimmun anti-S	NA	46 - 65	57% female, 43% male	supplement tables 1 + 2
51	10/15/2020	11/15/2020	10/30/2020	NA	Euroimmun anti-S	NA	46 - 65	57% female, 43% male	supplement tables 1 + 2
51	11/18/2020	12/15/2020	12/1/2020	NA	Euroimmun anti-S	NA	46 - 65	57% female, 43% male	supplement tables 1 + 2
51	10/14/2020	11/10/2020	10/27/2020	NA	Euroimmun anti-S	NA	46 - 65	57% female, 43% male	supplement tables 1 + 2
51	11/18/2020	12/15/2020	12/1/2020	NA	Euroimmun anti-S	NA	46 - 65	57% female, 43% male	supplement tables 1 + 2
51	1/25/2021	2/27/2021	2/10/2021	NA	Euroimmun anti-S	NA	46 - 65	57% female, 43% male	supplement tables 1 + 2
51	3/1/2021	3/31/2021	3/16/2021	NA	Euroimmun anti-S	NA	46 - 65	57% female, 43% male	supplement tables 1 + 2
51	4/7/2021	5/5/2021	4/21/2021	NA	Euroimmun anti-S	NA	46 - 65	57% female, 43% male	supplement tables 1 + 2

51	3/3/2021	8/17/2021	5/25/2021	NA	Euroimmun anti-S	NA	46 - 65	57% female, 43% male	supplement tables 1 + 2
51	5/12/2021	6/8/2021	5/25/2021	NA	Euroimmun anti-S	NA	46 - 65	57% female, 43% male	supplement tables 1 + 2
52 , 57	9/8/2020	9/26/2020	9/17/2020	NA	Euroimmun anti-S	NA	NA	NA	
53	NA	NA	NA	1	Euroimmun anti-S	NA	NA	NA	
53	NA	NA	NA	1 - 3	Euroimmun anti-S	NA	NA	NA	
53	NA	NA	NA	1	University of Rio de Janeiro	NA	NA	NA	
53	NA	NA	NA	1 - 3	University of Rio de Janeiro	NA	NA	NA	
54	4/6/2020	NA	NA	2	Euroimmun anti-S	2.9% hospitalized	44	54% female , 46% male	figure 1
54	NA	NA	NA	4	Euroimmun anti-S	2.9% hospitalized	44	54% female , 46% male	figure 1
54	NA	NA	NA	7	Euroimmun anti-S	2.9% hospitalized	44	54% female , 46% male	figure 1
54	NA	12/17/2020	NA	9	Euroimmun anti-S	2.9% hospitalized	44	54% female , 46% male	figure 1
57	6/23/2020	7/4/2020	6/28/2020	NA	Euroimmun anti-S	NA	NA	NA	
57	11/17/2020	12/5/2020	11/26/2020	NA	Euroimmun anti-S	NA	NA	NA	
58	NA	NA	11/11/2020	2	Euroimmun anti-S	NA	35 - 49	47% female , 53% male	table 1
59	6/20/2020	7/13/2020	7/1/2020	NA	Fortress Diagnostics	NA	NA	NA	
60	7/31/2020	8/13/2020	8/6/2020	NA	Fortress Diagnostics	NA	NA	NA	
60	9/15/2020	9/28/2020	9/21/2020	NA	Fortress Diagnostics	NA	NA	NA	
61	10/27/2020	11/10/2020	11/3/2020	NA	Fortress Diagnostics	NA	NA	NA	
62	4/27/2020	5/29/2020	5/13/2020	NA	Hangzhou Clongene	NA	NA	NA	
63	NA	NA	October 2020	8	THL Finland, in-house FMIA	13% hospitalized	49	57% female , 43% male	tables 1 and 2

63	NA	NA	May 2021	13	THL Finland, in-house FMIA	13% hospitalized	49	57% female , 43% male	tables 1 and 2
63	NA	NA	October 2020	8	THL Finland, in-house neutralisation assay	13% hospitalized	49	57% female , 43% male	tables 1 and 2
63	NA	NA	May 2021	13	THL Finland, in-house neutralisation assay	13% hospitalized	49	57% female , 43% male	tables 1 and 2
64	NA	NA	NA	4	THL Finland, in-house neutralisation assay	NA	NA	NA	
65	early November 2020	December 2020	NA	>6	THL Finland, in-house neutralisation assay	NA	NA	NA	
66	11/4/2020	1/22/2021	12/13/2020	NA	Gauteng, in-house Luminex	NA	NA	NA	
67	March 2021	April 2021		NA	Tunis, in-house	NA	NA	NA	
68	5/4/2020	5/13/2020	5/8/2020	NA	Livzon lateral flow	NA	NA	NA	
69	10/2/2020	10/11/2020	10/6/2020	1	Livzon lateral flow	NA	49 [34 - 59]	61% female, 39% male	table 2
69	10/2/2020	10/11/2020	10/6/2020	2	Livzon lateral flow	NA	49 [34 - 59]	61% female, 39% male	table 2
69	10/2/2020	10/11/2020	10/6/2020	4	Livzon lateral flow	NA	49 [34 - 59]	61% female, 39% male	table 2
69	10/2/2020	10/11/2020	10/6/2020	6	Livzon lateral flow	NA	49 [34 - 59]	61% female, 39% male	table 2
69	10/2/2020	10/11/2020	10/6/2020	7	Livzon lateral flow	NA	49 [34 - 59]	61% female, 39% male	table 2
70	4/9/2021	4/25/2021	4/17/2021	NA	Luminex xMAP (RBD portion)	NA	NA	NA	
71	11/5/2020	12/31/2020	12/3/2020	NA	Massachusetts General Hospital	NA	NA	NA	
72	October 2020	March 2021		NA	Mount Sinai (New York)	≤ 6% hospitalized	NA	NA	
72	October 2020	March 2021		>6	Mount Sinai (New York)	≤ 27% hospitalized	NA	NA	

73	5/18/2020	6/1/2020	5/25/2020	NA	Orient Gene Biotech	NA	NA	58% female , 42% male	tabla 2 on page 10
73	6/8/2020	6/22/2020	6/15/2020	NA	Orient Gene Biotech	NA	NA	58% female , 42% male	tabla 2 on page 10
74	11/16/2020	11/29/2020	11/22/2020	NA	Orient Gene Biotech	NA	NA	54% female , 46% male	tabla 2 on page 11
75	October 2020	February 2021	December 2020	NA	SenASTrIS test	NA	NA	NA	
76	5/4/2020	6/27/2020	5/31/2020	2 - 3	SenASTrIS test	15% hospitalized	49	56% female , 44% male	table 1
77	9/8/2020	10/14/2020	9/26/2020	NA	Siemens Advia Centaur	NA	NA	NA	
78	6/28/2020	7/9/2020	7/3/2020	NA	Standard Q	NA	NA	NA	
79	NA	NA	NA	2 - 3	Sinai Health (Toronto)	~26% hospitalized	58	48% female , 52% male	table 1, figure 1 legend
80	12/7/2020	1/15/2021	12/26/2020	NA	Techno Genetics	NA	NA	NA	
81	6/10/2020	7/29/2020	7/4/2020	NA	Vitros Ortho (not total antibodies)	NA	NA	NA	
82	1/11/2021	1/22/2021	1/16/2021	NA	Vitros Ortho (not total antibodies)	NA	NA	NA	
83	9/24/2021	10/14/2021	10/4/2021	NA	Vitros Ortho (not total antibodies)	NA	NA	NA	
84	10/15/2020	11/16/2020	10/31/2020	4	Vitros Ortho (not total antibodies)	NA	NA	NA	
84	4/18/2020	4/19/2020	4/18/2020	1	Wantai	NA	NA	NA	
86	5/18/2020	12/16/2020	9/1/2020	2	Wantai	NA	NA	NA	
87	2/27/2021	4/20/2021	3/17/2021	4	Wantai	NA	NA	NA	
88	5/14/2021	7/11/2021	6/10/2021	5	Wantai	NA	NA	NA	

B) Supplementary methods, statistical modeling

Cross validation posterior sample generation

Because the use of our estimates to predict sensitivity for serosurveys in the literature may involve extrapolating our curves through time (e.g. if an assay has data up to a limited time range), we used a cross-validation procedure that shows model performance with extrapolation through time. We achieved this by grouping the validation data points so that validation involved extrapolation through time.

First we selected an assay to be validated. Then, we computed the average of the times from diagnosis to serological testing reported across the assay's data points. We next separated the assay's data points into those with times earlier, and those with times later than the average time (i.e. cohorts serologically tested closer to their diagnosis date, and further to their diagnosis, respectively). We then fitted the model to all the data, except the data points with earlier times. We used this model fit to generate predictions of the sensitivities of the excluded data points (i.e. estimate the sensitivity of a given assay, at a given time). We repeated the procedure fitting all the data except the "later" data points for this assay (we now included the "earlier" data points for this assay in the model fit). We repeated this procedure for all assays, thus generating predictions for all the data points included in the analysis.

For the generation of the posterior samples, say that we want to estimate the number of positive tests $x_{a,s,t}$ for a left out data point corresponding to assay a, in study s, at time t. Suppose that other data points of the same assay a and of the same study s as this data point were used in model fitting. Then, at each posterior draw of the model, we have samples of the mean intercept and slope across assays, μ and β, samples of the assay specific intercept and slope effects u_a and b_a, and a sample of the study specific effect u_s. Then, we plug these parameter samples into equation (2) of the Methods in the main text, to obtain an assay and study specific sample of sensitivity at time t, $\theta_{a,s,t}$. Next, we use this sample of $\theta_{a,s,t}$ to proceed as explained in the main methods, sampling $x_{a,s,t}$ 5 times from binomial distribution in equation (1) with parameters $\theta_{a,s,t}$ and $N_{a,s,t}$. After repeating this procedure for all posterior samples of the parameters, we take the 2.5% and 97.5% quantiles of the $x_{a,s,t}$ samples as the 95% CrI of our prediction.

In the case of $x_{a,s,t}$ where no other sample from the same study was used in model fitting, we do not have posterior draws of the study-specific effect u_s generated by the model. For these cases, at each draw, we sample a value of u_s from the distribution $N(u_s|0, \sigma_{us})$, using the corresponding draw of σ_{us}, and then follow the procedure described above. Similarly, in the cases where $x_{a,s,t}$ is the only data point for an assay and there are no assay-specific effects u_a and b_a in the posterior sample, we sample these from $N(u_a|0, \sigma_{ua})$ and $N(b_a|0, \sigma_{ba})$ respectively. That is, for assays with only one data point, we only sample sensitivities from the overall distribution of sensitivities.

To obtain model predictions, we first sampled the sensitivity of a given data point from the fitted Bayesian model as described above. Then, for each posterior draw of sensitivity, we drew 5 samples of the number of positive tests using the sampled sensitivity and Equation 1 of the main text. This produced a posterior distribution of the number of positive tests for each data point.

We apply an analogous procedure to cross-validate the models that include effects of assay characteristics.

Estimation of times between diagnosis and serology

When studies did not report the median time between diagnosis and serological testing for their cohort, we estimated these times using reported case curves for the study's location. For this, we first took the cumulative number of reported cases *C* at the study's location at the date of serological sampling, and set the median date of COVID-19 diagnosis for the tested cohort as the date in which the cumulative cases were *C*/2 in this location. If the study's cohort is representative of the general population, the population's median diagnosis date should match that of the cohort. We then took the time elapsed between this median diagnosis date and the median serosurvey sampling date as the time from diagnosis to sampling for this data point. Case numbers were obtained from (90), or from data made available by public health authorities.

C) Assay-specific sensitivity profiles

With the models fitted to sensitivity data, we obtained the posterior probability of time-varying sensitivity across tests. **Figure S1** shows the estimated time-dependent sensitivity for each assay, the fitted data (each point in **Figure S1** corresponds to a row in **Table S2**), and we indicate which data points were within the 95% CrI of their cross-validated prediction. We show the sensitivity profiles predicted from the model without assay characteristics (black) and with assay characteristics (red). We also present the values estimated for time-varying sensitivity across 14 months for each assay in **Table S3** (i.e. the values corresponding to the red lines and their credible intervals).

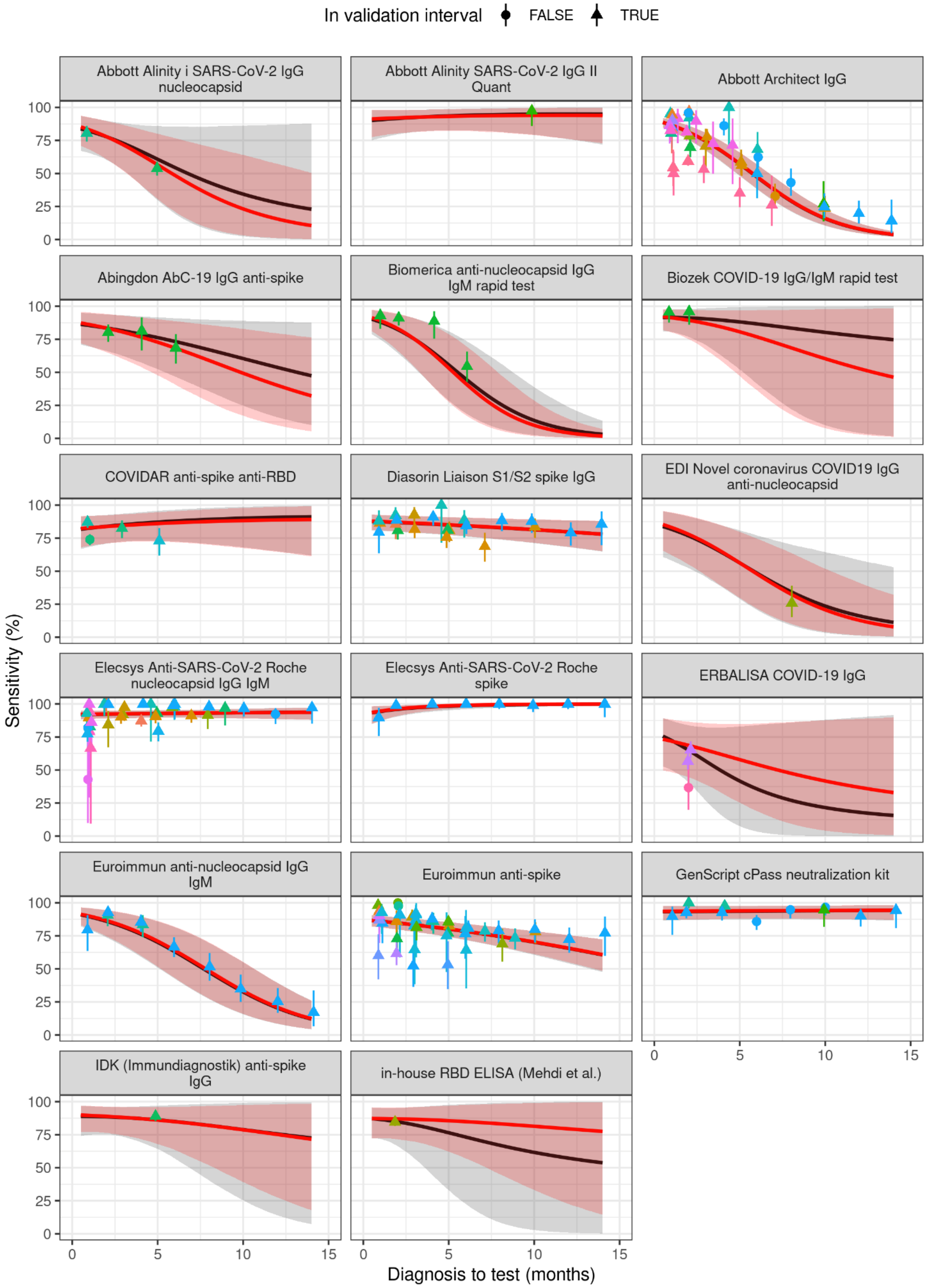

In validation interval FALSE TRUE
Abbott Alinity i SARS-CoV-2 IgG nucleocapsid
Abbott Alinity SARS-CoV-2 IgG II Quant
Abbott Architect IgG
Abingdon AbC-19 IgG anti-spike
Biomerica anti-nucleocapsid IgG IgM rapid test
Biozek COVID-19 IgG/IgM rapid test
COVIDAR anti-spike anti-RBD
Diasorin Liaison S1/S2 spike IgG
EDI Novel coronavirus COVID19 IgG anti-nucleocapsid
Elecsys Anti-SARS-CoV-2 Roche nucleocapsid IgG IgM
Elecsys Anti-SARS-CoV-2 Roche spike
ERBALISA COVID-19 IgG
Euroimmun anti-nucleocapsid IgG IgM
Euroimmun anti-spike
GenScript cPass neutralization kit
IDK (Immundiagnostik) anti-spike IgG
in-house RBD ELISA (Mehdi et al.)
Sensitivity (%)
Diagnosis to test (months)
0
25
50
75
100
0
5
10
15

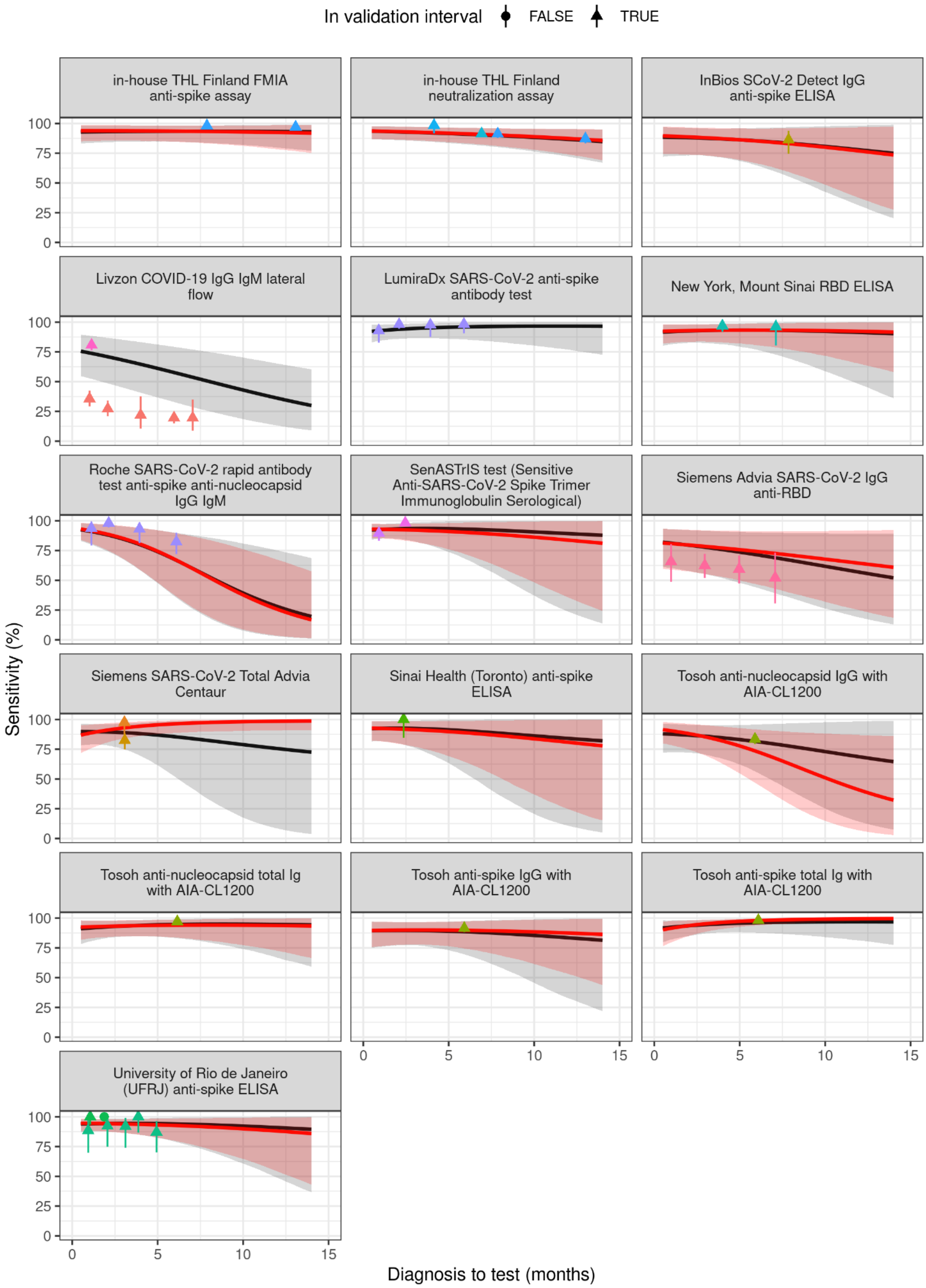

In validation interval
FALSE
TRUE
in-house THL Finland FMIA anti-spike assay
in-house THL Finland neutralization assay
InBios SCoV-2 Detect IgG anti-spike ELISA
Livzon COVID-19 IgG IgM lateral flow
LumiraDx SARS-CoV-2 anti-spike antibody test
New York, Mount Sinai RBD ELISA
Roche SARS-CoV-2 rapid antibody test anti-spike anti-nucleocapsid IgG IgM
SenASTrIS test (Sensitive Anti-SARS-CoV-2 Spike Trimer Immunoglobulin Serological)
Siemens Advia SARS-CoV-2 IgG anti-RBD
Siemens SARS-CoV-2 Total Advia Centaur
Sinai Health (Toronto) anti-spike ELISA
Tosoh anti-nucleocapsid IgG with AIA-CL1200
Tosoh anti-nucleocapsid total Ig with AIA-CL1200
Tosoh anti-spike IgG with AIA-CL1200
Tosoh anti-spike total Ig with AIA-CL1200
University of Rio de Janeiro (UFRJ) anti-spike ELISA
Sensitivity (%)
0
25
50
75
100
0
5
10
15
Diagnosis to test (months)

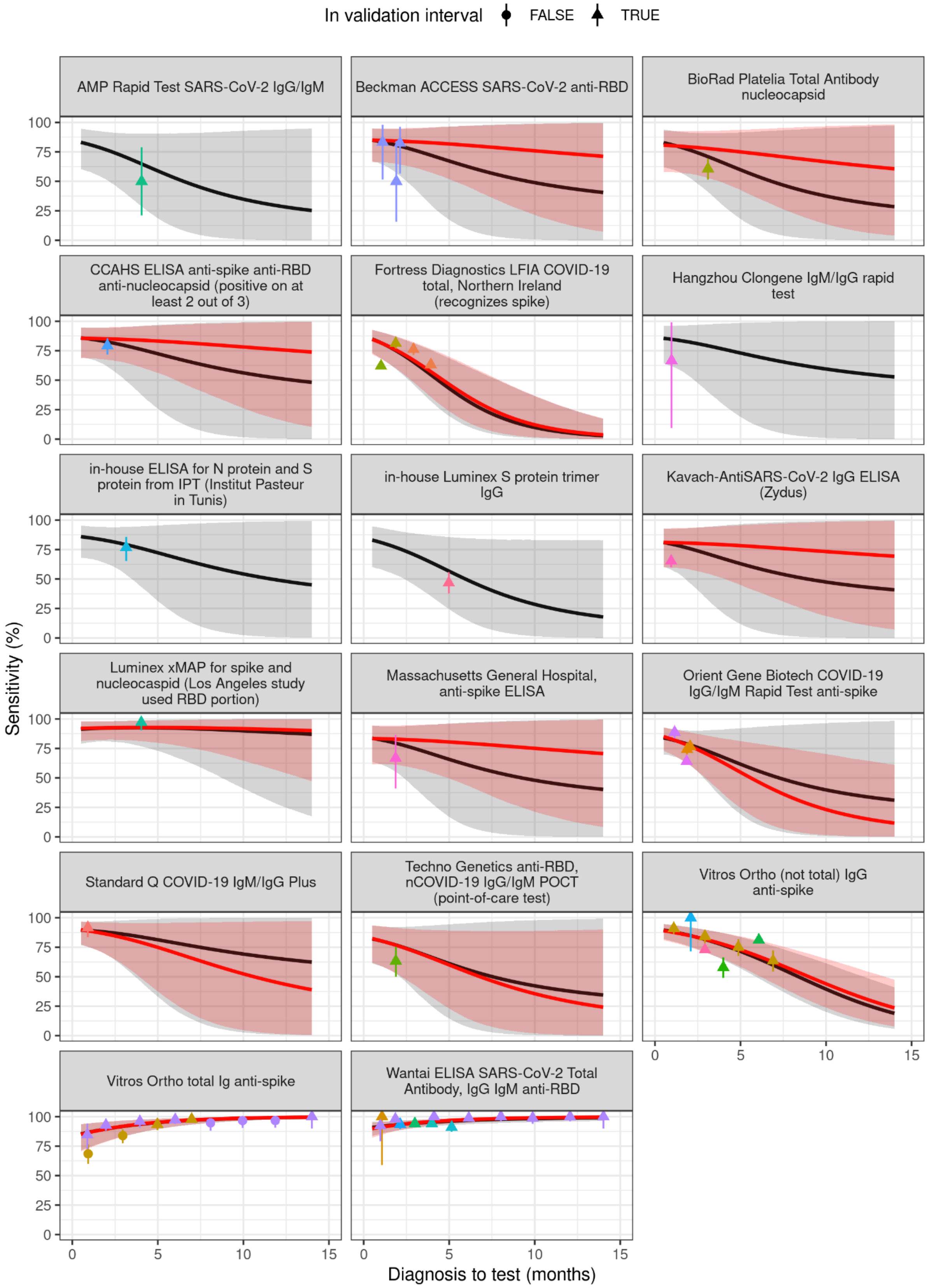

Figure S1. Assay-specific sensitivity profiles. Sensitivity across time estimated for all fitted assays. Each panel shows a different antibody assay. Points show the fitted data for each assay,

with vertical bars showing the 95% CI of each point. Point shape indicates whether the data point was inside their prediction interval in cross-validation (triangles) or not (circles). Black lines show the mean estimated sensitivity for each assay using the model without assay characteristics. The gray shaded region indicates the 95% posterior interval for the sensitivity estimates. Red lines and shaded regions show the same for the full model (taking into account assay antigen and analytical technique). Assays lacking a red line were not included in the full model fit, due to the absence of assay characteristics data. The color of the data points indicates the study that reported the data point, for easier identification of data points belonging to the same study.

Table S3. Time-varying sensitivity estimates for each assay. In this table we show the estimated sensitivities and 95% CrI (for the average assay sensitivity, not counting for between-study variability) for each assay included in our analysis. For assays that had sufficient information to fit the full model (e.g. do not lack information about the targeted antigen), we report the sensitivities obtained from the full model. If we lacked information about some assay characteristic, we report the sensitivities estimated with a model fitted without that characteristic. Assays that were not fit in the full model are indicated by an asterisk (*) after their name.

Assay	Month													
	1	2	3	4	5	6	7	8	9	10	11	12	13	14
Abbott Alinity i	83 (68-92)	78 (61-90)	72 (52-87)	64 (41-83)	57 (31-80)	49 (21-77)	41 (14-73)	34 (9-70)	28 (5-67)	23 (3-64)	19 (2-61)	16 (1-57)	13 (1-54)	11 (0-50)
Abbott Quant II	92 (79-98)	92 (80-98)	93 (81-98)	93 (82-98)	93 (82-99)	93 (82-99)	94 (82-99)	94 (81-99)	94 (80-99)	94 (79-99)	94 (77-100)	94 (76-100)	94 (75-100)	94 (73-100)
Abbott Architect	87 (82-90)	81 (76-86)	75 (68-81)	67 (58-74)	57 (49-66)	48 (39-57)	38 (29-47)	29 (22-38)	22 (15-30)	16 (11-23)	11 (7-17)	8 (5-12)	6 (3-9)	4 (2-6)
Abingdon AbC-19	86 (70-95)	83 (65-94)	80 (60-93)	77 (54-92)	73 (48-90)	68 (41-89)	64 (34-87)	59 (28-86)	54 (22-84)	49 (17-83)	45 (13-81)	40 (10-79)	36 (7-78)	32 (5-76)
Beckman ACCESS	85 (66-95)	84 (65-95)	83 (62-95)	83 (57-96)	82 (52-96)	81 (46-97)	80 (39-97)	78 (33-98)	77 (27-98)	76 (22-99)	75 (17-99)	73 (13-99)	72 (10-99)	71 (7-99)
Biomerica	89 (77-97)	83 (67-94)	75 (54-91)	65 (40-86)	53 (28-79)	41 (18-70)	31 (10-60)	22 (6-48)	15 (3-38)	10 (2-29)	6 (1-21)	4 (1-15)	3 (0-10)	2 (0-7)
BioRad Platelia	80 (58-93)	79 (57-92)	78 (53-93)	77 (49-93)	75 (43-94)	73 (36-94)	72 (29-95)	70 (23-96)	68 (18-96)	67 (14-97)	65 (10-97)	63 (7-98)	62 (5-98)	61 (4-98)
Biozek	91 (80-98)	89 (75-97)	87 (67-97)	84 (58-97)	81 (47-97)	77 (37-97)	73 (26-97)	69 (18-97)	64 (12-97)	60 (8-98)	56 (5-98)	53 (3-98)	49 (2-98)	46 (1-98)

CCAHS ELISA	85 (69-95)	85 (67-95)	85 (65-95)	84 (61-96)	83 (56-96)	82 (50-97)	81 (44-98)	80 (38-98)	79 (32-98)	78 (26-99)	77 (21-99)	76 (17-99)	75 (13-99)	74 (10-99)
COVIDAR	83 (69-92)	84 (71-92)	85 (72-93)	85 (72-94)	86 (72-95)	87 (72-95)	87 (71-96)	88 (70-97)	88 (69-97)	88 (67-98)	89 (66-98)	89 (65-99)	89 (63-99)	89 (61-99)
DiaSorin	88 (81-93)	87 (80-92)	86 (79-92)	86 (79-91)	85 (78-91)	84 (77-91)	84 (75-90)	83 (74-90)	82 (73-90)	81 (71-89)	81 (70-89)	80 (68-89)	79 (67-88)	78 (65-88)
Epitope Diagnostics, Inc.	83 (62-94)	78 (55-92)	72 (47-88)	64 (38-84)	56 (30-80)	48 (22-75)	40 (15-69)	33 (10-64)	26 (6-58)	21 (4-52)	16 (2-46)	13 (1-41)	10 (1-36)	8 (1-32)
Roche Elecsys N	92 (89-95)	92 (89-95)	93 (89-95)	93 (89-95)	93 (90-95)	93 (90-95)	93 (90-96)	93 (89-96)	93 (89-96)	93 (89-96)	93 (89-97)	94 (89-97)	94 (88-97)	94 (88-97)
Roche Elecsys S	95 (87-98)	96 (91-99)	97 (93-99)	98 (95-100)	99 (96-100)	99 (97-100)	99 (98-100)	99 (98-100)	100 (99-100)	100 (99-100)	100 (99-100)	100 (99-100)	100 (99-100)	100 (99-100)
ErbaLisa	72 (48-88)	69 (44-87)	65 (39-85)	62 (32-85)	58 (25-85)	54 (19-85)	51 (14-86)	48 (9-86)	44 (6-87)	42 (4-88)	39 (3-88)	37 (2-89)	35 (1-89)	33 (1-90)
Euroimmun N	90 (80-96)	87 (75-94)	82 (68-92)	77 (60-90)	71 (52-86)	64 (43-82)	56 (35-77)	48 (28-70)	41 (21-63)	33 (16-55)	27 (12-47)	21 (9-39)	16 (6-32)	12 (4-26)
Euroimmun S1	86 (81-90)	85 (80-89)	83 (78-88)	82 (76-87)	80 (74-85)	78 (72-84)	77 (70-83)	75 (67-81)	73 (64-80)	70 (61-79)	68 (58-77)	66 (55-76)	63 (52-74)	61 (48-72)
Fortress Diagnostics	82 (68-91)	75 (59-87)	66 (47-82)	56 (35-76)	46 (24-70)	37 (15-63)	29 (9-56)	22 (5-50)	16 (3-43)	12 (2-37)	9 (1-31)	7 (0-26)	5 (0-22)	4 (0-18)
GenScript cPass	94 (87-98)	94 (87-98)	94 (88-98)	94 (88-98)	94 (88-98)	94 (88-98)	94 (88-98)	94 (88-98)	94 (88-98)	94 (88-98)	94 (88-98)	94 (88-98)	94 (87-98)	94 (87-98)
Immundiagnostik	90 (77-97)	89 (76-96)	88 (74-96)	87 (72-96)	86 (68-96)	85 (64-96)	83 (58-96)	82 (52-97)	80 (45-97)	79 (39-97)	77 (33-98)	75 (28-98)	73 (22-98)	72 (18-98)

Mehdi, in-house	87 (72-95)	87 (72-96)	87 (70-96)	86 (67-96)	86 (62-97)	85 (57-97)	84 (51-98)	83 (45-98)	82 (39-99)	81 (33-99)	80 (28-99)	79 (23-99)	78 (18-99)	78 (14-100)
THL Finland FMIA	94 (85-99)	94 (86-99)	94 (86-98)	94 (85-98)	94 (85-98)	94 (85-98)	93 (84-98)	93 (83-98)	93 (82-98)	93 (81-98)	93 (80-98)	92 (78-98)	92 (77-99)	92 (75-99)
THL Finland neutralization	93 (87-98)	93 (86-97)	93 (86-97)	92 (85-97)	92 (84-97)	91 (83-96)	91 (82-96)	90 (81-96)	90 (79-96)	89 (78-96)	88 (76-95)	87 (74-95)	87 (72-95)	86 (69-95)
InBios SCoV-2 Detect	89 (74-97)	89 (74-97)	88 (73-96)	87 (72-96)	86 (69-96)	85 (66-96)	84 (63-96)	83 (59-96)	81 (54-96)	80 (49-96)	78 (43-97)	77 (37-97)	75 (32-97)	74 (28-97)
Zydus Kavach ELISA IgG	81 (60-93)	80 (58-93)	80 (55-94)	79 (51-95)	78 (45-96)	77 (39-96)	76 (34-97)	75 (28-98)	74 (23-98)	73 (19-98)	72 (15-99)	71 (12-99)	70 (9-99)	69 (7-99)
Luminex xMAP, RBD portion	92 (82-98)	92 (82-98)	93 (82-98)	93 (81-98)	93 (80-99)	93 (78-99)	93 (75-99)	92 (72-99)	92 (69-99)	92 (65-100)	91 (60-100)	91 (56-100)	91 (52-100)	90 (47-100)
Massachusetts General Hospital	83 (62-94)	83 (61-94)	82 (58-95)	81 (54-95)	80 (50-96)	79 (44-97)	78 (38-97)	77 (31-98)	76 (26-98)	75 (21-98)	74 (17-99)	73 (14-99)	72 (11-99)	71 (8-99)
Mount Sinai (New York)	93 (83-98)	93 (83-98)	93 (84-98)	93 (83-98)	93 (82-99)	93 (81-99)	93 (79-99)	93 (77-99)	93 (75-99)	93 (72-99)	93 (68-100)	92 (65-100)	92 (62-100)	92 (58-100)
Orient Gene	83 (70-91)	77 (61-89)	70 (49-86)	63 (36-83)	55 (24-81)	47 (15-79)	40 (9-77)	33 (5-75)	28 (3-72)	23 (1-70)	19 (1-68)	16 (0-65)	14 (0-63)	12 (0-61)

Biotech														
Roche, rapid test, S + N	92 (82-98)	89 (76-97)	85 (68-96)	80 (58-94)	74 (48-92)	68 (37-90)	60 (28-87)	52 (20-84)	45 (13-80)	38 (9-76)	31 (6-72)	26 (3-67)	21 (2-62)	17 (1-58)
SenASTrIS test	93 (85-97)	93 (84-97)	92 (82-98)	92 (80-98)	91 (76-98)	90 (72-98)	89 (67-99)	88 (61-99)	87 (55-99)	86 (48-99)	85 (42-99)	84 (36-100)	82 (30-100)	81 (24-100)
Siemens Advia SARS-CoV-2 IgG anti-RBD	81 (59-93)	80 (58-92)	78 (55-92)	77 (53-92)	76 (50-91)	74 (47-91)	73 (43-91)	71 (39-91)	69 (36-91)	68 (32-91)	66 (28-92)	64 (25-92)	63 (22-92)	61 (19-92)
Siemens Advia Centaur, Total	88 (75-96)	91 (80-97)	93 (84-98)	94 (86-98)	95 (88-99)	96 (89-99)	97 (89-100)	97 (90-100)	98 (90-100)	98 (90-100)	98 (91-100)	98 (91-100)	99 (91-100)	99 (91-100)
Sinai Health (Toronto) ELISA, S	92 (82-98)	92 (80-98)	91 (77-98)	91 (74-99)	90 (69-99)	89 (64-99)	88 (58-99)	86 (51-99)	85 (44-99)	84 (37-99)	82 (30-99)	81 (25-100)	79 (20-100)	78 (15-100)
SD Biosensor, Standard Q, Plus	89 (75-96)	86 (69-96)	83 (60-96)	79 (49-96)	75 (37-96)	70 (27-96)	66 (18-96)	61 (12-96)	57 (8-96)	52 (5-97)	49 (3-97)	45 (2-97)	42 (1-97)	39 (1-97)
Techno Genetics, anti-RBD POCT	81 (59-93)	76 (52-91)	71 (43-90)	66 (32-90)	60 (23-89)	54 (15-89)	49 (10-89)	44 (6-89)	40 (3-89)	36 (2-89)	32 (1-89)	29 (1-89)	26 (0-90)	24 (0-90)
Tosoh N indirect	90 (78-97)	88 (75-96)	85 (70-95)	82 (63-94)	78 (55-93)	73 (46-92)	68 (37-90)	62 (28-90)	57 (21-89)	51 (15-88)	46 (10-88)	41 (7-87)	36 (4-86)	32 (3-86)

Tosoh N sandwich	93 (83-98)	93 (84-98)	93 (85-98)	94 (85-98)	94 (85-98)	94 (84-99)	94 (83-99)	94 (81-99)	94 (79-99)	94 (77-99)	94 (75-100)	94 (73-100)	94 (70-100)	93 (66-100)
Tosoh S indirect	90 (76-97)	90 (78-97)	90 (78-97)	90 (77-97)	90 (76-97)	90 (74-97)	90 (72-98)	89 (69-98)	89 (66-98)	88 (62-99)	88 (57-99)	87 (53-99)	87 (49-99)	86 (44-99)
Tosoh S sandwich	92 (80-98)	94 (85-98)	95 (89-99)	96 (91-99)	97 (93-99)	98 (94-100)	98 (95-100)	99 (96-100)	99 (96-100)	99 (97-100)	99 (97-100)	99 (97-100)	100 (98-100)	100 (98-100)
UFRJ (University of Rio de Janeiro)	94 (88-98)	94 (88-98)	94 (86-98)	94 (85-98)	93 (83-98)	93 (80-98)	92 (77-99)	91 (73-99)	91 (69-99)	90 (65-99)	89 (60-99)	88 (54-99)	87 (49-99)	86 (43-100)
Vitros Ortho, S CoV2 IgG	88 (79-94)	85 (75-92)	82 (70-90)	77 (65-87)	73 (58-84)	68 (51-81)	62 (44-77)	56 (37-74)	50 (30-69)	44 (24-65)	38 (18-61)	33 (14-56)	28 (11-52)	24 (8-47)
Vitros Ortho, RBD CoV2T total	87 (73-94)	90 (79-96)	92 (83-97)	94 (87-98)	95 (89-98)	96 (92-99)	97 (93-99)	98 (95-99)	98 (96-99)	99 (97-100)	99 (97-100)	99 (98-100)	99 (98-100)	100 (99-100)
Wantai ELISA	91 (84-95)	93 (88-96)	95 (91-97)	96 (93-98)	97 (94-99)	97 (95-99)	98 (96-99)	98 (96-100)	99 (97-100)	99 (97-100)	99 (98-100)	99 (98-100)	99 (98-100)	100 (98-100)
AMP, rapid test (*)	82 (60-94)	74 (49-91)	66 (36-88)	56 (24-85)	46 (14-82)	38 (7-79)	30 (4-76)	24 (2-74)	19 (1-71)	16 (0-68)	13 (0-66)	11 (0-63)	9 (0-60)	7 (0-58)
Clungene (*)	81 (55-95)	74 (42-94)	66 (27-93)	57 (15-92)	49 (8-91)	41 (4-91)	35 (2-90)	30 (1-90)	26 (0-90)	22 (0-90)	19 (0-90)	17 (0-89)	15 (0-89)	14 (0-89)
Livzon (*)	74 (52-89)	71 (48-87)	67 (43-85)	63 (39-83)	60 (35-81)	56 (31-78)	52 (27-76)	48 (23-73)	44 (20-70)	40 (17-67)	37 (14-65)	33 (12-62)	30 (10-59)	27 (8-56)

Tunis, in-house (*)	85 (67-94)	85 (67-95)	84 (65-95)	84 (59-96)	83 (52-97)	82 (44-98)	81 (36-98)	80 (28-99)	79 (22-99)	78 (17-99)	77 (12-100)	76 (9-100)	75 (6-100)	74 (4-100)
Gauteng, in-house Luminex (*)	79 (52-92)	76 (51-91)	74 (48-90)	71 (43-90)	67 (35-90)	64 (28-91)	60 (20-91)	57 (14-92)	54 (9-93)	51 (6-94)	49 (4-95)	46 (3-95)	44 (2-96)	42 (1-96)
LumiraDx (*)	93 (84-98)	94 (86-98)	95 (87-99)	96 (88-99)	96 (88-99)	97 (88-100)	97 (88-100)	97 (88-100)	98 (88-100)	98 (87-100)	98 (87-100)	98 (86-100)	98 (86-100)	98 (85-100)

D) Assay sensitivity increase can continue across months

One somewhat surprising result of the analyses in the main text is that several assays show positive slopes that can be interpreted as an increase of sensitivity throughout the months. This is surprising because while antibody levels rise in the early stages after infection, they tend to stabilize or drop afterwards (82,83). Although previous work has also reported increased sensitivity across the early months after infection for some assays (9,10,84), we wondered whether it is possible that after an initial increase in sensitivity for these assays, then sensitivity stabilizes or drops. We tested this possibility by fitting a new model with two slopes, an initial slope up to 3 months after infection, and a later slope afterwards. First, we filtered out assays that did not have sensitivity estimates both before and after the cutoff point. Then, out of the remaining assays, we fitted this model to the 7 assays that target RBD, and that do not use the LFA technique, since they show the most positive group slopes in **Figure 2** in the main text.

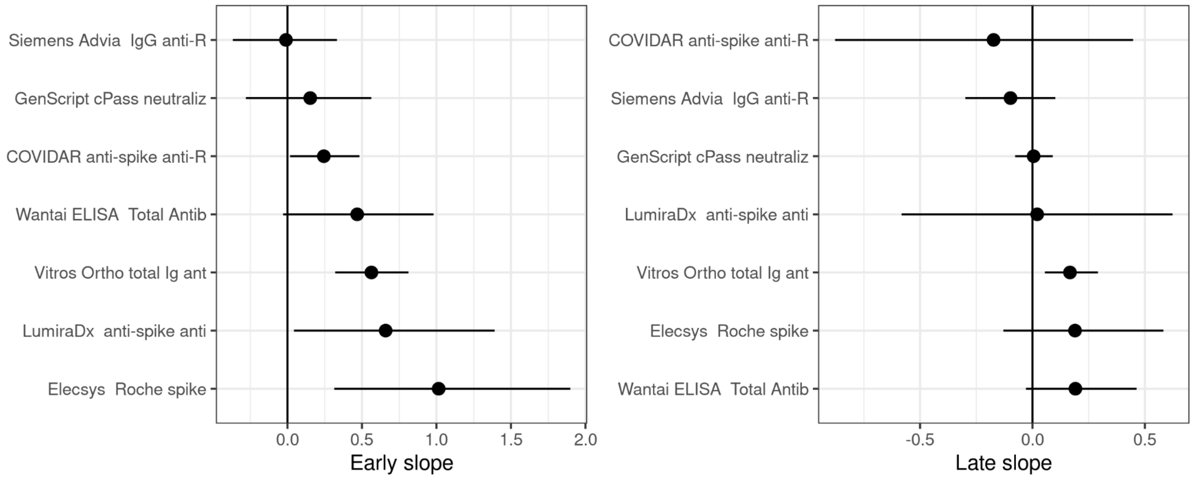

Figure S2. Values of early and late slopes. Values of the slope between 0 and 3 months (left panel) and the slope from 3 months onwards (right panel) for non-LFA assays that target RBD. Points show the mean of the posterior distribution of the parameters, and horizontal bars show the 95% credible interval (CrI).

In the model with two slopes, we obtained an average early slope (from 0 to 3 months) of 0.44 (CrI95%: 0.08; 0.87), and an average late slope (3 months onwards) of 0.04 (CrI95%: -0.33; 0.39). The later slope was smaller than the earlier slope in 93.6% of the posterior samples. This supports the idea that the increase in sensitivity mostly occurs during the early stages post infection. However, some assays seem to maintain a positive slope even after 3 months post infection (**Figure S1**). For example, for the assays *Elecsys Anti-SARS-CoV-2 Roche spike*, *Vitros*

Ortho total Ig anti-spike, and *Wantai ELISA SARS-CoV-2 Total Antibody anti-RBD* assays, the assay-specific later slopes was larger than 0 in 86.4%, 99.8%, and 95.2% of the posterior samples. Thus, our analysis shows that some assays can show an increase in sensitivity even several months after initial infection, although it is expected that sensitivity drops eventually for these assays, given enough time after infection. Finally, we note that although one could think that reinfections may drive this increase in sensitivity, we find this possibility to be unlikely. On the one hand, reinfections are unlikely to be common in the first 3 months after infection in seropositive individuals (91), where we do observe a positive slope for the Quantitative-Direct RBD-targeting assays. Then, many of the studies were performed during 2020, when reinfections were still relatively uncommon.

E) Robustness to model architecture

To examine the robustness of the model presented in the main text, considering all relevant assay characteristics simultaneously, we analyze here each assay characteristic separately.

We first fitted the model containing only the terms for assay antigen. That is, the model had a different slope for each antigen. The posterior distributions of these parameters are shown in **Figure S3A**, and the sensitivity profiles are shown in **Figure S3B**. We find that N<S in 83.4% of the posterior samples (as suggested by **Figure S3A**). The mean slope of RBD had a positive value of 0.09 [-0.07; 0.23] with RBD>0 in 84.3% of the posterior samples. Also, RBD had the slowest seroreversion, with RBD>S in 98.2% of the posterior samples, RBD>N in 99.8% of the samples. These results are in agreement with the results reported in the main text, with the difference of showing a more significant difference between RBD and S targeting assays.

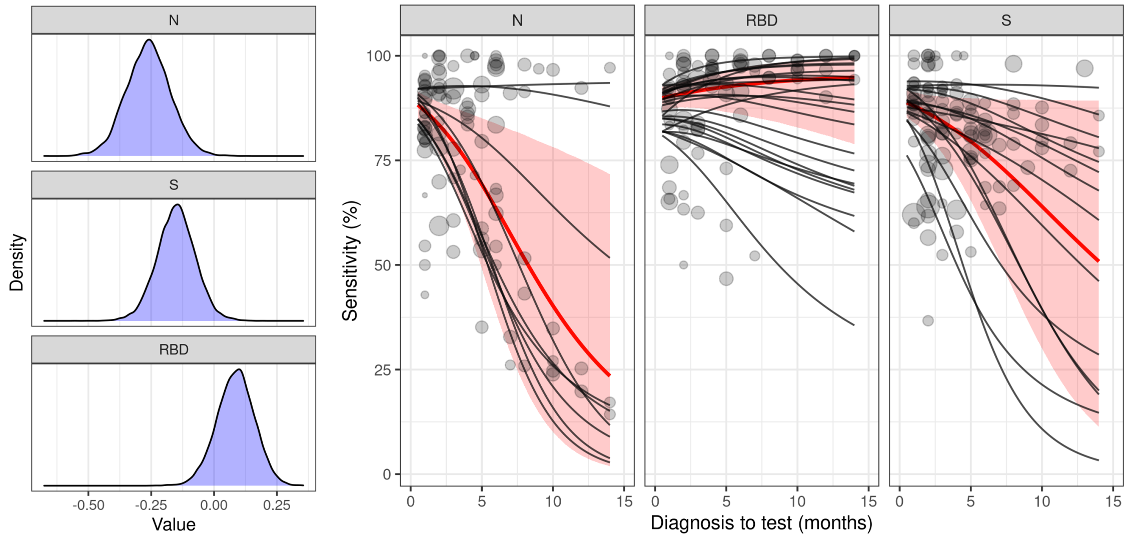

Figure S3. Effect of antigen on sensitivity. Estimated seroreversion segregated by the antigen used in the antibody assay. **A)** Posterior distribution of the effects of each antigen on the sensitivity slope. **B)** The sensitivity profile across time for each kind of assay is shown in a different panel. Panel titles indicate what slope parameters are added together to obtain the slope of each type of assay. The red lines show the mean sensitivity for each kind of assay across time, and the shaded regions show the 95% CrI. Note that the 95% CrI refer to the uncertainty over the mean sensitivity, and they do not account for the variability between assays. Gray lines show the fits for individual assays. Gray dots show the data, with dot size proportional to the square root of the number of samples.

Next, we looked at the effect of assay analytical technique. In particular, we fitted a different slope to each different type of analytic technique: LFA, Quantiative-Indirect, Quantitative-Direct and Competitive. In **Figure S4A** we see that the slopes for the different types of assays. The LFA slope had a value of -0.35 [-0.53; -0.19], the Quantitative-Indirect had a slope value of -0.12 [-0.22; -0.01], the Quantititative-Direct had a slope value of 0.20 [0.02; 0.38], and the Quantitative-Competitive had a slope value of -0.02 [-0.29; 0.25]. In **Figure S4B** we see the sensitivity across time profiles for these two kinds of assays. Similar to the results reported in the main text, we find that LFA assays have on average much faster resoreversion that quantitative assays. Also, within the quantitative assays, those using the direct binding design have more positive slopes, while there is considerable uncertainty in the model around the slope of assays using competitive binding (which in this case are neutralization assays).

We note that in further analysis (not shown), we did not find a clear difference between assays using ELISA, CLIA and CMIA types of detection method within the Quantitative assays.

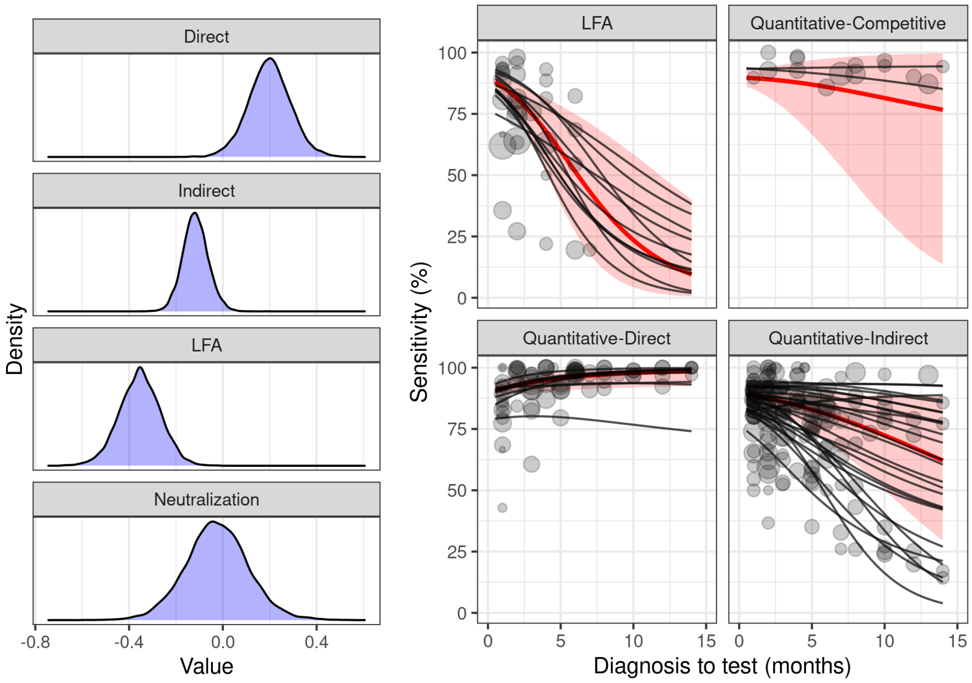

Figure S4. Effect of analytic technique on sensitivity. A) Posterior distribution of the mean slopes for each analytic technique. **B)** Sensitivity across time for each analytic technique. Same conventions as **Figure S3B**.

F) Robustness to time estimation

In our main analysis, we estimated the time between diagnosis and testing for samples where this number was unknown (see Methods). To test the robustness of the results to this procedure, we repeated the main analysis including only samples where the time between diagnosis and serology testing was known.

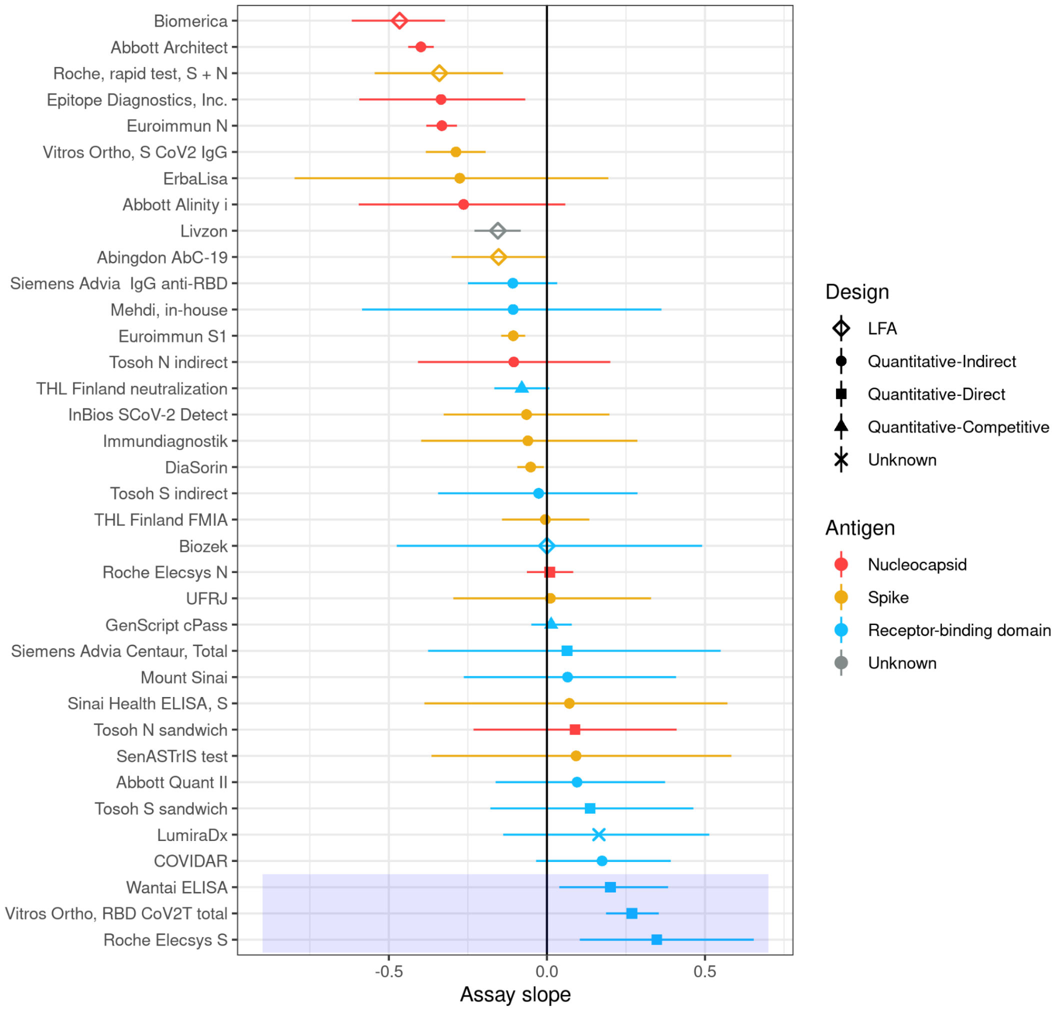

Figure S5. Assay slopes estimated without assay characteristics, for samples with known times. Same as **Figure 2** in the main text, but excluding the samples where the times between diagnosis and serology testing were estimated.

In **Figure S5** we see the slopes of the individual assays, analogous to **Figure 2** in the main text, and in **Figure S6** we see the average slopes for the different types of assays, analogous to **Figure 3** in the main text. We see that fewer assays are included in this analysis, because some assays only had data with unknown diagnosis to testing times. However, we see that the overall trends reported in the main text with the full dataset are the same as the ones obtained here with

only known diagnosis to testing times. Estimated model parameters and statistical analyses are also in line with the main results (results not shown).

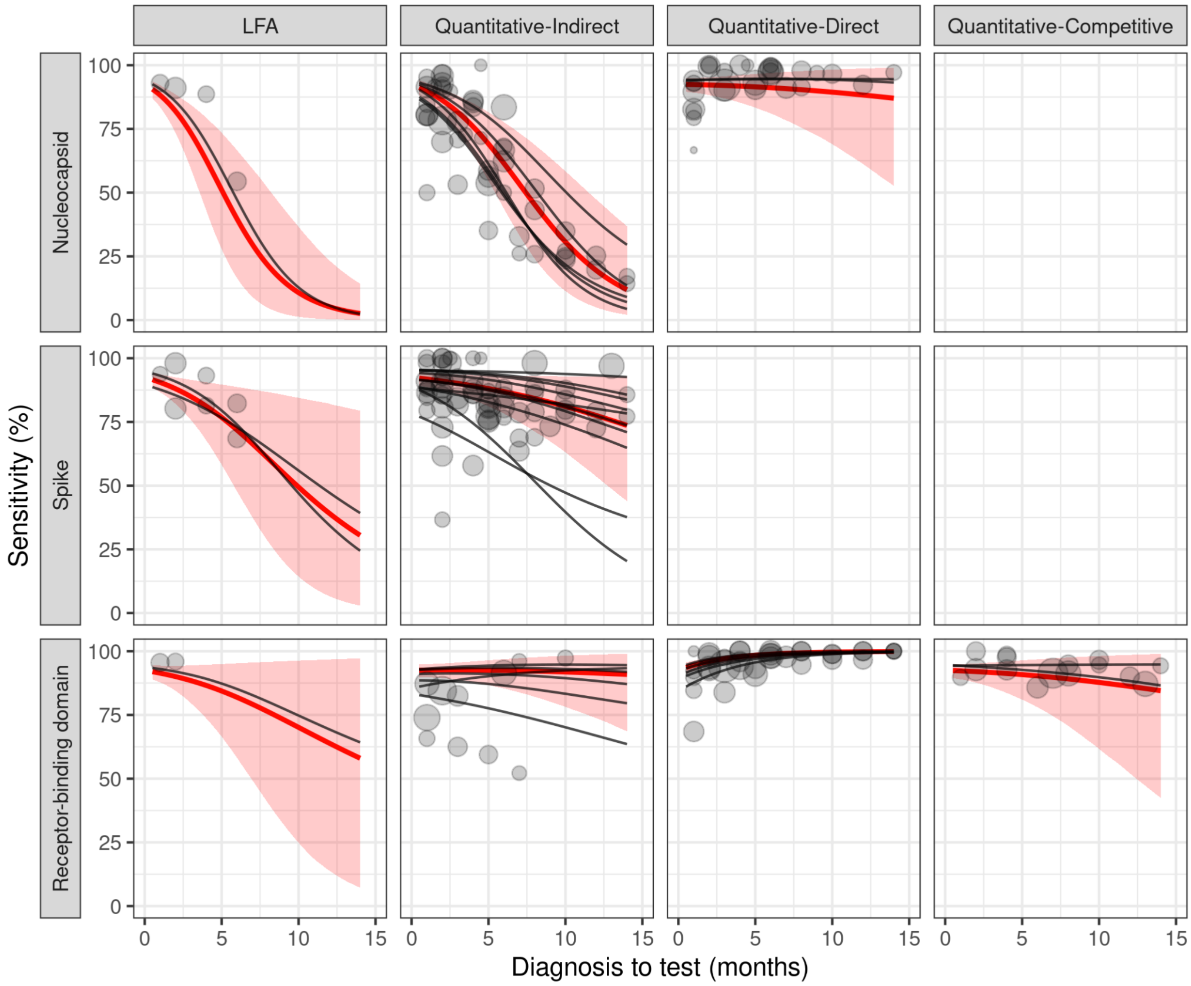

Figure S6. Sensitivity profiles for different assay characteristics, for samples with known times. Same as **Figure 3** in the main text, but excluding the samples where the times between diagnosis and serology testing were estimated.

G) Effect of assay characteristics on specificity

We fitted a hierarchical Bayesian model to specificity data reported for the assays included in our main analysis. Because assay specificity is not expected to have relevant dynamics (as opposed to sensitivity), we fitted only the point estimates of specificity reported for each assay by either the manufacturer, or independent studies. We fitted a model analogous to the model with assay characteristics discussed in the main text, with some modifications.

First, in the binomial distribution $P(x|\theta) \propto \theta^{x} (1 - \theta)^{N-x}$ used to model the raw data, N corresponds to the number of tested negative samples (e.g. pre-pandemic samples), and x corresponds to the number of negative samples (i.e. the true negatives). In this context, parameter θ corresponds to assay specificity.

Then, we used the following formula to model average specificity by assay characteristics:

$$log\left(\frac{\theta_{a,s}}{1-\theta_{a,s}}\right) = \left(u_a + u_s + \beta_{LFA}L_a + \beta_{Direct}D_a + \beta_{Competitive}C_a + \beta_{Nucleocapsid}N_a + \beta_{Spike}S_a + \beta_{RBD}RBD_a\right)$$

In this model, we do not have a temporal component, all terms only affect the point value of specificity. We have an assay specific term u_a, a study specific term u_s, and the terms indicating the effects of different assay characteristics (e.g. β_{LFA} the effect of being a qualitative, LFA assay), all analogous to the model described for sensitivity in the main text (e.g. we omit the Indirect assay term, making it the baseline specificity). Priors of the model parameters and the fitting procedure were set as for the model in the main text.

In **Figure S7** we see the raw specificity data, as well as the results obtained from the model fit. We obtained that LFA assays have on average lower specificity than Quantitative-Indirect assays (β_{LFA}<0 in 98.4% of the posterior samples), but we did not find a significant effect for the Quantitative-Direct design (β_{Direct}>0 in 85.0% of the posterior samples), the Quantitative-Competitive design ($\beta_{Competitive}$<0 in 86.6% of the posterior samples), or for the antigen targeted (β_{Spike}>$\beta_{Nucleocapsid}$ in 32.3% of the posterior samples, β_{RBD}>$\beta_{Nucleocapsid}$ in 67.3% of the posterior samples). Estimated sensitivities for different assay types are shown in **Table S4**.

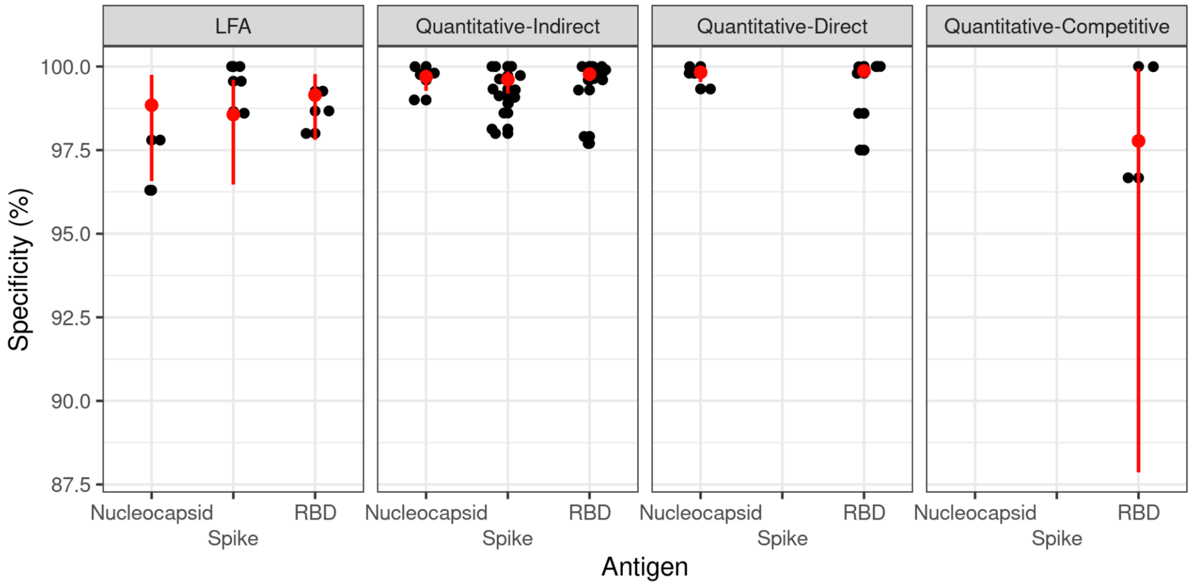

Figure S7. Assay specificity for different assay characteristics. Each black dot shows the specificity reported for a given assay, by a given study. Different panels indicate different test design, and the horizontal axis within each panel indicates the different antigens. Red dots and vertical lines indicate the mean sensitivity and 95% credible interval as obtained from the hierarchical Bayesian model fitted to the specificity data.

Table S4. Mean specificity and 95% CrI for the different types of assays, obtained from the fitted Bayesian model. Credible intervals do not account for variability between assays of a same type, or within studies. Values correspond to the red dots and lines in **Figure S7**.

**Design**	**Antigen**	**Specificity % (95% CrI)**
LFA	N	98.8 (96.6-99.7)
LFA	S	98.6 (96.5-99.6)
LFA	RBD	99.1 (97.8-99.8)
Quantitative-Indirect	N	99.7 (99.3-99.9)
Quantitative-Indirect	S	99.6 (99.1-99.9)
Quantitative-Indirect	RBD	99.8 (99.6-99.9)
Quantitative-Direct	N	99.8 (99.5-99.9)
Quantitative-Direct	RBD	99.8 (99.7-100)
Quantitative-Competitive	RBD	97.8 (87.9-99.9)

References

1. van den Hoogen LL, Smits G, van Hagen CCE, Wong D, Vos ERA, van Boven M, et al. Seropositivity to Nucleoprotein to detect mild and asymptomatic SARS-CoV-2 infections: A complementary tool to detect breakthrough infections after COVID-19 vaccination? Vaccine. 2022 Apr 1;40(15):2251–7.
2. Lohse S, Sternjakob-Marthaler A, Lagemann P, Schöpe J, Rissland J, Seiwert N, et al. German federal-state-wide seroprevalence study of 1st SARS-CoV-2 pandemic wave shows importance of long-term antibody test performance. Commun Med. 2022 May 18;2(1):1–12.
3. Silveira MF, Mesenburg M, Dellagostin OA, Oliveira NR, Maia MAC, Santos FDS, et al. Time-Dependent Decay of Detectable Antibodies Against SARS-CoV-2: A Comparison of ELISA with Two Batches of a Lateral-Flow Test [Internet]. Rochester, NY: Social Science Research Network; 2021 Jan [cited 2021 Jul 26]. Report No.: ID 3757411. Available from: https://papers.ssrn.com/abstract=3757411
4. McCance K, Wise H, Simpson J, Batchelor B, Hale H, McDonald L, et al. Evaluation of SARS-CoV-2 antibody point of care devices in the laboratory and clinical setting. PLOS ONE. 2022 Mar 31;17(3):e0266086.
5. Kobak D. Underdispersion: A statistical anomaly in reported Covid data. Significance. 2022;19(2):10–3.
6. Mallon PWG, Tinago W, Leon AG, McCann K, Kenny G, McGettrick P, et al. Dynamic Change and Clinical Relevance of Postinfectious SARS-CoV-2 Antibody Responses. Open Forum Infect Dis. 2021 Aug 1;8(8):ofab122.
7. Eberhardt KA, Dewald F, Heger E, Gieselmann L, Vanshylla K, Wirtz M, et al. Evaluation of a New Spike (S)-Protein-Based Commercial Immunoassay for the Detection of Anti-SARS-CoV-2 IgG. Microorganisms. 2021 Apr;9(4):733.
8. McGregor R, Craigie A, Jack S, Upton A, Moreland NJ, Ussher J. The Persistence of Neutralising Antibodies up to 11 months after SARS-CoV-2 Infection in the Southern Region of New Zealand [Internet]. medRxiv; 2021 [cited 2022 Sep 7]. p. 2021.10.26.21265501. Available from: https://www.medrxiv.org/content/10.1101/2021.10.26.21265501v1
9. Peluso MJ, Takahashi S, Hakim J, Kelly JD, Torres L, Iyer NS, et al. SARS-CoV-2 antibody magnitude and detectability are driven by disease severity, timing, and assay. Sci Adv. 2021 Jul 30;7(31):eabh3409.
10. Scheiblauer H, Nübling CM, Wolf T, Khodamoradi Y, Bellinghausen C, Sonntagbauer M, et al. Antibody response to SARS-CoV-2 for more than one year − kinetics and persistence of detection are predominantly determined by avidity progression and test design. J Clin Virol. 2022 Jan 1;146:105052.
11. Bond KA, Williams E, Nicholson S, Lim S, Johnson D, Cox B, et al. Longitudinal evaluation of laboratory-based serological assays for SARS-CoV-2 antibody detection. Pathology (Phila). 2021 Oct 1;53(6):773–9.
12. Knabl L, Mitra T, Kimpel J, Rössler A, Volland A, Walser A, et al. High SARS-CoV-2 seroprevalence in children and adults in the Austrian ski resort of Ischgl. Commun Med. 2021 Jun 30;1(1):1–9.
13. Pollán M, Pérez-Gómez B, Pastor-Barriuso R, Oteo J, Hernán MA, Pérez-Olmeda M, et al. Prevalence of SARS-CoV-2 in Spain (ENE-COVID): a nationwide, population-based seroepidemiological study. The Lancet. 2020 Aug 22;396(10250):535–44.
14. Samore MH, Looney A, Orleans B, Greene T, Seegert N, Delgado JC, et al. Probability-Based Estimates of Severe Acute Respiratory Syndrome Coronavirus 2 Seroprevalence and Detection Fraction, Utah, USA. Emerg Infect Dis. 2021 Nov;27(11):2786–94.
15. Tadesse EB, Endris AA, Solomon H, Alayu M, Kebede A, Eshetu K, et al. Seroprevalence and risk factors for SARS-CoV-2 Infection in selected urban areas in

Ethiopia: a cross-sectional evaluation during July 2020. Int J Infect Dis. 2021 Oct 1;111:179–85.
16. Murhekar MV, Bhatnagar T, Selvaraju S, Saravanakumar V, Thangaraj JWV, Shah N, et al. SARS-CoV-2 antibody seroprevalence in India, August–September, 2020: findings from the second nationwide household serosurvey. Lancet Glob Health. 2021 Mar 1;9(3):e257–66.
17. Ayub T, Raja MW, Khan SMS, Haq I, Qureshi MA, Majid S, et al. Seroprevalence of SARS CoV 2 specific Ig G antibodies in District Srinagar, Kashmir: a population based study. Int J Community Med Public Health. 2021 Mar 25;8(4):1792.
18. Khan SMS, Qurieshi MA, Haq I, Majid S, Ahmad J, Ayub T, et al. Seroprevalence of SARS-CoV-2-specific IgG antibodies in Kashmir, India, 7 months after the first reported local COVID-19 case: results of a population-based seroprevalence survey from October to November 2020. BMJ Open. 2021 Sep 1;11(9):e053791.
19. Murhekar MV, Bhatnagar T, Thangaraj JWV, Saravanakumar V, Kumar MS, Selvaraju S, et al. Seroprevalence of IgG antibodies against SARS-CoV-2 among the general population and healthcare workers in India, June–July 2021: A population-based cross-sectional study. PLOS Med. 2021 Dec 10;18(12):e1003877.
20. Murhekar MV, Bhatnagar T, Thangaraj JWV, Saravanakumar V, Kumar MS, Selvaraju S, et al. SARS-CoV-2 seroprevalence among the general population and healthcare workers in India, December 2020–January 2021. Int J Infect Dis. 2021 Jul 1;108:145–55.
21. Thiruvengadam R, Chattopadhyay S, Mehdi F, Desiraju BK, Chaudhuri S, Singh S, et al. Longitudinal Serology of SARS-CoV-2-Infected Individuals in India: A Prospective Cohort Study. Am J Trop Med Hyg. 2021 Jul 7;105(1):66–72.
22. Tea F, Stella AO, Aggarwal A, Darley DR, Pilli D, Vitale D, et al. SARS-CoV-2 neutralizing antibodies: Longevity, breadth, and evasion by emerging viral variants. PLOS Med. 2021 Jul 6;18(7):e1003656.
23. Garritsen A, Scholzen A, van den Nieuwenhof DWA, Smits APF, Datema ES, van Galen LS, et al. Two-tiered SARS-CoV-2 seroconversion screening in the Netherlands and stability of nucleocapsid, spike protein domain 1 and neutralizing antibodies. Infect Dis. 2021 Jul 3;53(7):498–512.
24. Muecksch F, Wise H, Batchelor B, Squires M, Semple E, Richardson C, et al. Longitudinal Serological Analysis and Neutralizing Antibody Levels in Coronavirus Disease 2019 Convalescent Patients. J Infect Dis. 2021 Feb 1;223(3):389–98.
25. Kahre E, Galow L, Unrath M, Haag L, Blankenburg J, Dalpke AH, et al. Kinetics and seroprevalence of SARS-CoV-2 antibodies: a comparison of 3 different assays. Sci Rep. 2021 Jul 21;11(1):14893.
26. Kumar MS, Thangaraj JWV, Saravanakumar V, Selvaraju S, Kumar CPG, Sabarinathan R, et al. Monitoring the trend of SARS-CoV-2 seroprevalence in Chennai, India, July and October 2020. Trans R Soc Trop Med Hyg. 2021 Nov 1;115(11):1350–2.
27. Šmigelskas K, Petrikonis K, Kasiulevičius V, Kalėdienė R, Jakaitienė A, Kaselienė S, et al. SARS-CoV-2 Seroprevalence in Lithuania: Results of National Population Survey. Acta Medica Litu. 2021;28(1):48–58.
28. Kennedy JL, Forrest JC, Young SG, Amick B, Williams M, James L, et al. Temporal Variations in Seroprevalence of Severe Acute Respiratory Syndrome Coronavirus 2 Infections by Race and Ethnicity in Arkansas. Open Forum Infect Dis. 2022 May 1;9(5):ofac154.
29. Sullivan PS, Siegler AJ, Shioda K, Hall EW, Bradley H, Sanchez T, et al. Severe Acute Respiratory Syndrome Coronavirus 2 Cumulative Incidence, United States, August 2020–December 2020. Clin Infect Dis. 2022 Apr 1;74(7):1141–50.
30. Bushnik T. COVID-19 infection in the Canadian household population. Health Rep. 2022;33(82):12.
31. Schonfeld D, Fernández H, Ramírez J, Acosta D, Becerra J, Wettstein M, et al. SARS-CoV-2 seroprevalence in the city of Puerto Madryn: Underdiagnosis and relevance of children in the pandemic. PLOS ONE. 2022 Mar 14;17(3):e0263679.
32. Ojeda DS, Ledesma MMGL, Pallarés HM, Navarro GSC, Sanchez L, Perazzi B, et al.

Emergency response for evaluating SARS-CoV-2 immune status, seroprevalence and convalescent plasma in Argentina. PLOS Pathog. 2021 Jan 14;17(1):e1009161.
33. Longueira Y, Polo ML, Group I working, Turk G, Laufer N, Group B de EICC working. Dynamics of SARS-CoV-2-specific antibodies during and after COVID19: Lessons from a biobank in Argentina [Internet]. medRxiv; 2021 [cited 2022 Sep 7]. p. 2021.01.31.21250167. Available from: https://www.medrxiv.org/content/10.1101/2021.01.31.21250167v1
34. Dorigatti I, Lavezzo E, Manuto L, Ciavarella C, Pacenti M, Boldrin C, et al. SARS-CoV-2 antibody dynamics and transmission from community-wide serological testing in the Italian municipality of Vo'. Nat Commun. 2021 Jul 19;12(1):4383.
35. Choe PG, Kim KH, Kang CK, Suh HJ, Kang E, Lee SY, et al. Antibody Responses 8 Months after Asymptomatic or Mild SARS-CoV-2 Infection. Emerg Infect Dis. 2021 Mar;27(3):928–31.
36. Kar SS, Sarkar S, Murali S, Dhodapkar R, Joseph NM, Aggarwal R. Prevalence and Time Trend of SARS-CoV-2 Infection in Puducherry, India, August–October 2020. Emerg Infect Dis. 2021 Feb;27(2):666–9.
37. Herlinda O, Bella A, Kusnadi G, Nurshadrina DS, Akbar MT, Nida S, et al. Seroprevalence of antibodies against SARS-Cov-2 in the high impacted sub-district in Jakarta, Indonesia. PLOS ONE. 2021 Dec 23;16(12):e0261931.
38. Laxmaiah A, Rao NM, Arlappa N, Babu J, Kumar PU, Singh P, et al. SARS-CoV-2 seroprevalence in the city of Hyderabad, India in early 2021. IJID Reg. 2022 Mar 1;2:1–7.
39. https://www.mhlw.go.jp/content/10900000/000934787.pdf [Internet]. https://www.mhlw.go.jp/content/10900000/000934787.pdf. [cited 2022 Sep 7]. Available from: https://www.mhlw.go.jp/content/10900000/000934787.pdf
40. Kshatri JS, Bhattacharya D, Kanungo S, Giri S, Palo SK, Parai D, et al. Serological surveys to inform SARS-CoV-2 epidemic curve: a cross-sectional study from Odisha, India. Sci Rep. 2021 May 18;11(1):10551.
41. Kshatri JS, Bhattacharya D, Praharaj I, Mansingh A, Parai D, Kanungo S, et al. Seroprevalence of SARS-CoV-2 in Bhubaneswar, India: findings from three rounds of community surveys. Epidemiol Infect [Internet]. 2021 ed [cited 2022 Apr 8];149. Available from: https://www.cambridge.org/core/journals/epidemiology-and-infection/article/seroprevalence-of-sarscov2-in-bhubaneswar-india-findings-from-three-rounds-of-community-surveys/29A7905F297A1EFA68749AC3C6383300
42. Gudbjartsson DF, Norddahl GL, Melsted P, Gunnarsdottir K, Holm H, Eythorsson E, et al. Humoral Immune Response to SARS-CoV-2 in Iceland. N Engl J Med. 2020 Oct 29;383(18):1724–34.
43. Goto A, Go H, Miyakawa K, Yamaoka Y, Ohtake N, Kubo S, et al. Sustained Neutralizing Antibodies 6 Months Following Infection in 376 Japanese COVID-19 Survivors. Front Microbiol [Internet]. 2021 [cited 2022 Sep 7];12. Available from: https://www.frontiersin.org/articles/10.3389/fmicb.2021.661187
44. Alkharaan H, Bayati S, Hellström C, Aleman S, Olsson A, Lindahl K, et al. Persisting Salivary IgG Against SARS-CoV-2 at 9 Months After Mild COVID-19: A Complementary Approach to Population Surveys. J Infect Dis. 2021 Aug 2;224(3):407–14.
45. Sharma N, Sharma P, Basu S, Saxena S, Chawla R, Dushyant K, et al. The seroprevalence of severe acute respiratory syndrome coronavirus 2 in Delhi, India: a repeated population-based seroepidemiological study. Trans R Soc Trop Med Hyg. 2022 Mar 2;116(3):242–51.
46. Satpati PS, Sarangi SS, Gantait KS, Endow S, Mandal NC, Panchanan K, et al. Sero-surveillance (IgG) of SARS-CoV-2 among Asymptomatic General population of Paschim Medinipur, West Bengal, India [Internet]. medRxiv; 2020 [cited 2022 Apr 8]. p. 2020.09.12.20193219. Available from: https://www.medrxiv.org/content/10.1101/2020.09.12.20193219v1
47. Warszawski J, Beaumont AL, Seng R, de Lamballerie X, Rahib D, Lydié N, et al.

Prevalence of SARS-Cov-2 antibodies and living conditions: the French national random population-based EPICOV cohort. BMC Infect Dis. 2022 Jan 9;22(1):41.
48. Carrat F, de Lamballerie X, Rahib D, Blanché H, Lapidus N, Artaud F, et al. Antibody status and cumulative incidence of SARS-CoV-2 infection among adults in three regions of France following the first lockdown and associated risk factors: a multicohort study. Int J Epidemiol. 2021 Oct 1;50(5):1458–72.
49. Santos-Hövener C, Neuhauser HK, Rosario AS, Busch M, Schlaud M, Hoffmann R, et al. Serology- and PCR-based cumulative incidence of SARS-CoV-2 infection in adults in a successfully contained early hotspot (CoMoLo study), Germany, May to June 2020. Eurosurveillance. 2020 Nov 26;25(47):2001752.
50. Ghose A, Bhattacharya S, Karthikeyan AS, Kudale A, Monteiro JM, Joshi A, et al. Community prevalence of antibodies to SARS-CoV-2 and correlates of protective immunity in an Indian metropolitan city [Internet]. medRxiv; 2020 [cited 2022 Sep 7]. p. 2020.11.17.20228155. Available from: https://www.medrxiv.org/content/10.1101/2020.11.17.20228155v2
51. Gornyk D, Harries M, Glöckner S, Strengert M, Kerrinnes T, Bojara G, et al. SARS-CoV-2 Seroprevalence in Germany - A Population Based Sequential Study in Five Regions [Internet]. Rochester, NY: Social Science Research Network; 2021 Apr [cited 2021 Dec 4]. Report No.: ID 3834300. Available from: https://papers.ssrn.com/abstract=3834300
52. RKI - CORONA-MONITORING lokal - Corona-Monitoring lokal [Internet]. [cited 2022 Sep 7]. Available from: https://www.rki.de/DE/Content/Gesundheitsmonitoring/Studien/cml-studie/Factsheet_Straubing.html
53. Alvim RGF, Lima TM, Rodrigues DAS, Marsili FF, Bozza VBT, Higa LM, et al. Development and large-scale validation of a highly accurate SARS-COV-2 serological test using regular test strips for autonomous and affordable finger-prick sample collection, transportation, and storage [Internet]. medRxiv; 2021 [cited 2022 Sep 7]. p. 2020.07.13.20152884. Available from: https://www.medrxiv.org/content/10.1101/2020.07.13.20152884v6
54. Vanshylla K, Cristanziano VD, Kleipass F, Dewald F, Schommers P, Gieselmann L, et al. Kinetics and correlates of the neutralizing antibody response to SARS-CoV-2 [Internet]. bioRxiv; 2021 [cited 2022 Sep 7]. p. 2021.01.26.428207. Available from: https://www.biorxiv.org/content/10.1101/2021.01.26.428207v1
55. Parrott JC, Maleki AN, Vassor VE, Osahan S, Hsin Y, Sanderson M, et al. Prevalence of SARS-CoV-2 Antibodies in New York City Adults, June–October 2020: A Population-Based Survey. J Infect Dis. 2021 Jul 15;224(2):188–95.
56. Ärzteblatt DÄG Redaktion Deutsches. SARS-CoV-2 Seroprevalence in Germany (03.12.2021) [Internet]. Deutsches Ärzteblatt. [cited 2023 Feb 14]. Available from: https://www.aerzteblatt.de/int/archive/article?id=221933
57. Seroepidemiologische Studie zur Abschätzung der Verbreitung von SARS-CoV-2 in der Bevölkerung an besonders betroffenen Orten in Deutschland [Internet]. Robert Kock Institut; 2021. Available from: https://www.rki.de/DE/Content/Gesundheitsmonitoring/Studien/cml-studie/Dokumente/Abschlussbericht_2021.pdf?__blob=publicationFile
58. Neuhauser H, Schaffrath Rosario A, Butschalowsky H, Haller S, Hoebel J, Michel J, et al. Germany's low SARS-CoV-2 seroprevalence confirms effective containment in 2020: Results of the nationwide RKI-SOEP study. medRxiv [Internet]. 2021 Nov 24; Available from: https://doi.org/10.1101/2021.11.22.21266711
59. Ward H, Atchison C, Whitaker M, Ainslie KEC, Elliott J, Okell L, et al. SARS-CoV-2 antibody prevalence in England following the first peak of the pandemic. Nat Commun. 2021 Feb 10;12(1):905.
60. Ward H, Cooke GS, Atchison C, Whitaker M, Elliott J, Moshe M, et al. Prevalence of antibody positivity to SARS-CoV-2 following the first peak of infection in England: Serial cross-sectional studies of 365,000 adults. Lancet Reg Health – Eur [Internet]. 2021 May

1 [cited 2022 Apr 8];4. Available from: https://www.thelancet.com/journals/lanepe/article/PIIS2666-7762(21)00075-2/fulltext
61. Ward H, Atchison C, Whitaker M, Donnelly CA, Riley S, Ashby D, et al. Increasing SARS-CoV-2 antibody prevalence in England at the start of the second wave: REACT-2 Round 4 cross-sectional study in 160,000 adults [Internet]. 2021 Jul [cited 2021 Dec 3] p. 2021.07.21.21260926. Available from: https://www.medrxiv.org/content/10.1101/2021.07.21.21260926v1
62. Iruzubieta P, Fernández-Lanas T, Rasines L, Cayon L, Álvarez-Cancelo A, Santos-Laso A, et al. Feasibility of large-scale population testing for SARS-CoV-2 detection by self-testing at home. Sci Rep. 2021 May 10;11(1):9819.
63. Haveri A, Ekström N, Solastie A, Virta C, Österlund P, Isosaari E, et al. Persistence of neutralizing antibodies a year after SARS-CoV-2 infection in humans. Eur J Immunol. 2021;51(12):3202–13.
64. Study by THL and Welfare and the City of Helsinki: Antibodies elicited by coronavirus infection persist for at least four months - Press release - THL [Internet]. Finnish Institute for Health and Welfare (THL), Finland. [cited 2022 Sep 7]. Available from: https://thl.fi/en/web/thlfi-en/-/study-by-thl-and-welfare-and-the-city-of-helsinki-antibodies-elicited-by-coronavirus-infection-persist-for-at-least-four-months
65. THL's study: Majority of people who have had a coronavirus infection retain antibodies for over six months - Press release - THL [Internet]. Finnish Institute for Health and Welfare (THL), Finland. [cited 2022 Sep 7]. Available from: https://thl.fi/en/web/thlfi-en/-/thl-s-study-majority-of-people-who-have-had-a-coronavirus-infection-retain-antibodies-for-over-six-months
66. Mutevedzi PC, Kawonga M, Kwatra G, Moultrie A, Baillie V, Mabena N, et al. Estimated SARS-CoV-2 infection rate and fatality risk in Gauteng Province, South Africa: a population-based seroepidemiological survey. Int J Epidemiol. 2022 Apr 1;51(2):404–17.
67. Cherif I, Kharroubi G, Chaabane S, Yazidi R, Dellagi M, Snoussi MA, et al. COVID-19 in Tunisia (North Africa): Seroprevalence of SARS-CoV-2 in the general population of the capital city Tunis [Internet]. In Review; 2022 Feb [cited 2022 Sep 7]. Available from: https://www.researchsquare.com/article/rs-1317280/v2
68. Royo-Cebrecos C, Vilanova D, López J, Arroyo V, Pons M, Francisco G, et al. Mass SARS-CoV-2 serological screening, a population-based study in the Principality of Andorra. Lancet Reg Health - Eur. 2021 Jun 1;5:100119.
69. Fogh K, Strange JE. Testing Denmark: a Danish Nationwide Surveillance Study of COVID-19. Microbiol Spectr. 9(3):12.
70. Sood N, Pernet O, Lam CN, Klipp A, Kotha R, Kovacs A, et al. Seroprevalence of Antibodies Specific to Receptor Binding Domain of SARS-CoV-2 and Vaccination Coverage Among Adults in Los Angeles County, April 2021: The LA Pandemic Surveillance Cohort Study. JAMA Netw Open. 2022 Jan 20;5(1):e2144258.
71. Matias WR, Fulcher IR, Sauer SM, Nolan CP, Guillaume Y, Zhu J, et al. Disparities in SARS-CoV-2 exposure: evidence from a citywide seroprevalence study in Holyoke, Massachusetts, USA [Internet]. medRxiv; 2021 [cited 2022 Sep 7]. p. 2021.10.13.21264975. Available from: https://www.medrxiv.org/content/10.1101/2021.10.13.21264975v1
72. Giuliano AR, Pilon-Thomas S, Schell MJ, Abrahamsen M, Islam JY, Isaacs-Soriano K, et al. SARS-CoV-2 Period Seroprevalence and Related Factors, Hillsborough County, Florida, USA, October 2020–March 2021. Emerg Infect Dis. 2022 Mar;28(3):556–63.
73. ESTUDIO ENE-COVID: INFORME FINAL ESTUDIO NACIONAL DE SERO-EPIDEMIOLOGÍA DE LA INFECCIÓN POR SARS-COV-2 EN ESPAÑA [Internet]. Ministerio de Sanidad, Gobierno de España; 2020. Available from: https://portalcne.isciii.es/enecovid19/informes/informe_final.pdf
74. ESTUDIO ENE-COVID: CUARTA RONDA ESTUDIO NACIONAL DE SERO-EPIDEMIOLOGÍA DE LA INFECCIÓN POR SARS-COV-2 EN ESPAÑA [Internet]. Ministerio de Sanidad, Gobierno de España; 2020. Available from: https://www.sanidad.gob.es/gabinetePrensa/notaPrensa/pdf/15.12151220163348113.pdf

75. Blankenberger J, Kaufmann M, Albanese E, Amati R, Anker D, Camerini AL, et al. Is living in a household with children associated with SARS-CoV-2 seropositivity in adults? Results from the Swiss national seroprevalence study Corona Immunitas. BMC Med. 2022 Jun 20;20(1):233.
76. Dupraz J, Butty A, Duperrex O, Estoppey S, Faivre V, Thabard J, et al. Prevalence of SARS-CoV-2 in Household Members and Other Close Contacts of COVID-19 Cases: A Serologic Study in Canton of Vaud, Switzerland. Open Forum Infect Dis. 2021 Jul 1;8(7):ofab149.
77. Castro LC e, Pereira AHG, Ribeiro R, Alves C, Veloso L, Vicente V, et al. Prevalence of SARS-CoV-2 Antibodies after First 6 Months of COVID-19 Pandemic, Portugal. Emerg Infect Dis [Internet]. 2021 Nov [cited 2022 Sep 7];27(11). Available from: https://wwwnc.cdc.gov/eid/article/27/11/21-0636_article
78. Reyes-Vega MF, Soto-Cabezas MG, Cárdenas F, Martel KS, Valle A, Valverde J, et al. SARS-CoV-2 prevalence associated to low socioeconomic status and overcrowding in an LMIC megacity: A population-based seroepidemiological survey in Lima, Peru. eClinicalMedicine [Internet]. 2021 Apr 1 [cited 2022 Sep 7];34. Available from: https://www.thelancet.com/journals/eclinm/article/PIIS2589-5370(21)00081-X/fulltext
79. Isho B, Abe KT, Zuo M, Jamal AJ, Rathod B, Wang JH, et al. Persistence of serum and saliva antibody responses to SARS-CoV-2 spike antigens in COVID-19 patients. Sci Immunol. 2020 Oct 8;5(52):eabe5511.
80. Hoballah A, El Haidari R, Siblany G, Abdel Sater F, Mansour S, Hassan H, et al. SARS-CoV-2 antibody seroprevalence in Lebanon: findings from the first nationwide serosurvey. BMC Infect Dis. 2022 Jan 10;22(1):42.
81. Mahajan S, Srinivasan R, Redlich CA, Huston SK, Anastasio KM, Cashman L, et al. Seroprevalence of SARS-CoV-2-Specific IgG Antibodies Among Adults Living in Connecticut: Post-Infection Prevalence (PIP) Study. Am J Med. 2021 Apr 1;134(4):526-534.e11.
82. Sharma N, Sharma P, Basu S, Bakshi R, Gupta E, Agarwal R, et al. Second Wave of the COVID-19 Pandemic in Delhi, India: High Seroprevalence Not a Deterrent? Cureus [Internet]. 2021 Oct 24 [cited 2022 Sep 7];13(10). Available from: https://www.cureus.com/articles/72911-second-wave-of-the-covid-19-pandemic-in-delhi-india-high-seroprevalence-not-a-deterrent
83. Sharma P, Basu S, Mishra S, Gupta E, Aggarwal R, Kale P, et al. SARS-CoV-2 seroprevalence in Delhi, India - September-October 2021 – a population based seroepidemiological study [Internet]. medRxiv; 2021 [cited 2022 Sep 7]. p. 2021.12.28.21268451. Available from: https://www.medrxiv.org/content/10.1101/2021.12.28.21268451v1
84. Abdul-Raheem R, Moosa S, Waheed F, Aboobakuru M, Ahmed IN, Rafeeg FN, et al. A sero-epidemiological study after two waves of the COVID-19 epidemic. Asian Pac J Allergy Immunol. 2021 Dec 26;
85. Cito F, Amato L, Di Giuseppe A, Danzetta ML, Iannetti S, Petrini A, et al. A COVID-19 Hotspot Area: Activities and Epidemiological Findings. Microorganisms. 2020 Nov;8(11):1711.
86. Espenhain L, Tribler S, Særke Jørgensen C, Holm Hansen C, Wolff Sönksen U, Ethelberg S. Prevalence of SARS-CoV-2 antibodies in Denmark: nationwide, population-based seroepidemiological study. Eur J Epidemiol [Internet]. 2021 Aug 22 [cited 2021 Aug 26]; Available from: https://doi.org/10.1007/s10654-021-00796-8
87. Covid-19: Den Nationale Prævalensundersøgelse Resultaterne fra 4. runde af antistofundersøgelse med 50.000 udtrukne borgere, uge 9-12, 2021 [Internet]. Statens Serum Institut; 2021. Available from: https://files.ssi.dk/praevalensundersoegelse_runde4
88. Covid-19: Den Nationale Prævalensundersøgelse Resultaterne fra 5. runde af prævalensundersøgelsen med 75.000 udtrukne borgere, uge 19-23, 2021. Statens Serum Institut; 2021.
89. Herrera-Esposito D, de los Campos G. Age-specific rate of severe and critical

SARS-CoV-2 infections estimated with multi-country seroprevalence studies. BMC Infect Dis. 2022 Mar 29;22(1):311.
90. Guidotti E, Ardia D. COVID-19 Data Hub. J Open Source Softw. 2020 Jul 10;5(51):2376.
91. Cromer D, Juno JA, Khoury D, Reynaldi A, Wheatley AK, Kent SJ, et al. Prospects for durable immune control of SARS-CoV-2 and prevention of reinfection. Nat Rev Immunol. 2021 Jun;21(6):395–404.
